# Maternal and Neonatal Complications Associated with Breast Cancer Systemic Treatments—A VigiBase Disproportionality Analysis Study

**DOI:** 10.1002/cpt.70404

**Published:** 2026-07-29

**Authors:** Rayan Kabirian, Floriane Jochum, Elise Dumas, Florence Coussy, Kevin Bihan, Bénédicte Lebrun‐Vignes, Barbara Pistilli, Marc‐Antoine Benderra, Lise Selleret, Laurent Chouchana, Fabien Reyal, Anne‐Sophie Hamy, Paul Gougis

**Affiliations:** ^1^ Residual Tumor & Response to Treatment Laboratory, RT2Lab, INSERM, U932 Immunity and Cancer Institut Curie, Université Paris Cité Paris France; ^2^ Institut Curie Women's Cancers Institute, Department of Medical Oncology Paris and Saint‐Cloud France; ^3^ Institute of Mathematics, Ecole Polytechnique Fédérale de Lausanne (EPFL) Lausanne Switzerland; ^4^ Residual Tumor & Response to Treatment Laboratory, RT2Lab, INSERM, U932 Immunity and Cancer, Institut Curie, Université Paris Cité Paris France; ^5^ Regional Center of Pharmacovigilance, Department of Medical Pharmacology, Pitié‐Salpêtrière Hospital, AP‐HP Sorbonne Université Paris France; ^6^ French Pharmacovigilance Network. Assistance Publique ‐ Hôpitaux de Paris (AP‐HP) Paris France; ^7^ Clinical Investigation Center (CIC‐1901) Institut National de la Santé et de la Recherche Médicale (INSERM) and Epidemiology in Dermatology and Evaluation of therapeutics), Université Paris Est Créteil (UPEC) Paris France; ^8^ Institut Gustave Roussy, Department of Medical Oncology Villejuif France; ^9^ Institut Universitaire de Cancérologie, Sorbonne University, AP‐HP, Tenon Hospital, Department of Medical Oncology Paris France; ^10^ Department of Obstetrics, Gynecology, and Reproductive Medicine Tenon Hospital, AP‐HP, Sorbonne Université Paris France; ^11^ Université Paris Cité, Inserm UMR‐S 1343, Pharmacologie et évaluations des thérapeutiques chez l'enfant et la femme enceinte Paris France; ^12^ Department of Breast, Gynecological and Reconstructive Surgery, Institut Curie Université Paris Paris France; ^13^ Sorbonne Université, Institut national de la santé et de la recherche médicale (INSERM), Assistance Publique ‐ Hôpitaux de Paris (AP‐HP), Clinical Investigation Center (CIC‐1901), Department of Pharmacology Pitié‐Salpêtrière Hospital Paris France; ^14^ Sorbonne Université, Department of Medical Oncology, Assistance Publique ‐ Hôpitaux de Paris (AP‐HP) Pitié‐Salpêtrière Hospital, Institut Universitaire de Cancérologie Paris France

## Abstract

Pregnancy‐associated breast cancer (PrBC) presents therapeutic challenges. Understanding maternal–fetal safety of systemic anticancer therapies is critical. We performed a case/non‐case disproportionality analysis using the WHO global pharmacovigilance database up to January 2024, to evaluate maternal–fetal outcomes associated with breast cancer (BC) systemic treatments. The exposure group consisted of reports mentioning a BC treatment at any time during pregnancy. The primary outcome was the reporting odds ratio (ROR) of maternal–fetal complications with the two cornerstone cytotoxic classes, anthracyclines (doxorubicin, epirubicin) and taxanes (docetaxel, paclitaxel), assessed individually vs. other anticancer treatments. Secondary analyses assessed all BC treatments and trimester‐specific risks. Of 3310 pregnancy‐related reports, 1789 involved BC treatments. Median maternal age was 27.4 years (SD 13.8) vs. 29.3 (SD 11.7) in the comparator group. Adverse maternal–fetal outcomes were reported in 998 (55.8%) BC treatment reports vs. 470 (30.9%) in other anticancer treatments. Anthracyclines were associated with neonatal hematologic disorders (ROR = 1.7, 95% CI = 1.1–2.5) and intrauterine growth restriction (IUGR) (ROR = 2.1, 95% CI = 1.7–2.7). Paclitaxel was linked to sensory defects including congenital ear/ocular anomalies (ROR = 5.2, 95% CI = 2.2–12.3); all 8 cases involved platinum co‐exposure (cisplatin: *n* = 5; carboplatin: *n* = 3), suggesting a platinum‐mediated mechanism; and to hyperbilirubinemia (ROR = 4.1, 95% CI = 2.0–8.4), and docetaxel to neonatal neurologic disorders (ROR = 3.6, 95% CI = 1.4–9.3). First‐trimester exposure (*n* = 26) showed preliminary signals of increased congenital malformation risk, including face (ROR = 13, 95% CI = 2–79), and musculoskeletal malformation (ROR = 8, 95% CI = 1.1–60). This pharmacovigilance study identifies signals of disproportionate reporting of specific maternal and neonatal outcomes with BC treatments; these findings are hypothesis‐generating and require confirmation in prospective registries.

**Study Highlights**

**WHAT IS THE CURRENT KNOWLEDGE ON THE TOPIC?**

Breast cancer during pregnancy is rare but increasing, and systemic chemotherapy, particularly anthracyclines and taxanes, is often required. Existing evidence suggests these agents can be administered during the second and third trimesters with manageable risks, while first‐trimester exposure is associated with higher rates of fetal toxicity. However, current knowledge is based on small cohorts, limiting drug‐specific safety assessment and comparisons within drug classes.

**WHAT QUESTION DID THIS STUDY ADDRESS?**

This study evaluated maternal and fetal/neonatal adverse outcomes associated with in utero exposure to commonly used breast cancer treatments, focusing on individual drug safety profiles within the two cornerstone cytotoxic classes, anthracyclines (doxorubicin, epirubicin) and taxanes (paclitaxel, docetaxel), and the impact of timing of exposure using a large global pharmacovigilance database.

**WHAT DOES THIS STUDY ADD TO OUR KNOWLEDGE?**

Using over 3,000 reports from VigiBase, this study provides large‐scale evidence of increased reporting of adverse pregnancy and neonatal outcomes with breast cancer treatments compared to other anticancer therapies. It identifies distinct toxicity patterns between drugs within the same class, including a novel signal of congenital malformations with epirubicin and vascular‐placental toxicity with taxanes. Sensitivity analyses confirm that the neonatal hematological disorder signal associated with anthracyclines is primarily driven by cyclophosphamide co‐administration rather than anthracyclines per se, and that key signals are robust to comparator group composition. The elevated risk associated with first‐trimester exposure is reinforced, though estimates remain preliminary given the small sample size (*n* = 26).

**HOW MIGHT THIS CHANGE CLINICAL PHARMACOLOGY OR TRANSLATIONAL SCIENCE?**

These findings support more individualized, drug‐specific risk assessment in pregnant patients with breast cancer and highlight the importance of treatment timing. They may guide regimen selection and scheduling relative to gestational age, while informing future pharmacokinetic and safety studies to better understand fetal effects of anticancer agents.

Breast cancer (BC) is the most common malignancy associated with pregnancy.^1^, ^2^ Clinical situations range from a diagnosis made during pregnancy to pregnancy occurring during ongoing cancer treatment.^3^, ^4^ It is estimated that 0.2–2.6% of all BCs are diagnosed during pregnancy,^5^ and up to 10% of BCs in women under 40 are pregnancy‐associated.^6^, ^7^ The incidence is rising, partly due to the global trend of delayed childbearing,^8^ making pregnancy‐associated breast cancer (PrBC) an increasingly important clinical issue.

The treatment of pregnant patients diagnosed with BC is a considerable challenge,^9^ requiring timely intervention while carefully balancing potential risks to the fetus, leading to complex benefit–risk assessments that encompass both maternal and fetal/neonatal outcomes. The importance of systemic therapies, particularly cytotoxic chemotherapy, has been established, along with the increased risks associated with first‐trimester exposure.^10^ However, current guidelines are based on limited data from small sample sizes, which precludes a detailed analysis of individual drugs and their precise safety profiles.^11^, ^12^ Some evidence suggests that certain drug classes, such as anthracyclines, alkylating agents, or taxanes, can be used safely during the second and third trimesters, with a moderate increase in certain maternofetal risks, such as the approximately twofold rise in intra‐uterine growth restriction reported with taxanes.^13^ Even within the same class, placental transfer can vary considerably.^14^ For example, docetaxel and paclitaxel are pharmacologically distinct^15^ and may have different maternofetal toxicity profiles, but no studies have adequately addressed these differences, highlighting a critical knowledge gap. Moreover, while the detrimental impact of first‐trimester exposure on fetal outcomes is well recognized, its precise effects have not yet been comprehensively described.

During pregnancy, breast cancer is most often diagnosed at a localized or locally advanced stage, and systemic chemotherapy is frequently administered in the neoadjuvant setting to control disease progression until delivery. As a result, anthracycline‐ and taxane‐based regimens constitute the backbone of systemic treatment in this context, underscoring the clinical importance of evaluating the specific maternal and fetal safety profiles of these agents.

VigiBase is the World Health Organization's global database that encompasses over 40 million individual case safety reports (ICSRs) from more than 180 countries; it offers an opportunity to assess rare but clinically significant adverse events across a diverse international population. We previously used VigiBase to evaluate the complications associated with exposure during pregnancy of several anticancer treatments and found that VigiBase can provide valuable insights into the fetal and maternal risks of these anticancer treatments.^16^, ^17^, ^18^

In this case/non‐case disproportionality analysis pharmacovigilance study, the primary objective was to describe the pre‐specified maternal and neonatal adverse outcomes reported following *in utero* doxorubicin and epirubicin (anthracyclines) and paclitaxel and docetaxel (taxanes), compared to other anticancer treatments in VigiBase. These four agents were selected because they represent the two guideline‐recommended cytotoxic classes for pregnancy‐associated BC, have the largest individual exposure counts in the database, and allow pharmacologically meaningful within‐class comparisons. Secondary objectives were to evaluate the impact of first‐trimester exposure on maternal and neonatal outcomes and to assess outcomes with all other BC treatments (including cyclophosphamide, fluorouracil, anti‐HER2 agents, and other targeted therapies).

## METHODS

### Study design

In this cohort study, we used safety reports from VigiBase, the WHO's global database of reported potential side effects of medicinal products, maintained by the Uppsala Monitoring Centre.^19^ A case/non‐case disproportionality analysis was conducted to examine the association between maternal and fetal/newborn adverse outcomes and exposure to BC treatments compared to other anticancer treatments used during pregnancy.

### Data query and report extraction

VigiBase was queried on January 1st, 2024, using Medical Dictionary for Regulatory Activities (MedDRA) version 26.1. We extracted de‐duplicated VigiMatch individual case safety reports (hereafter referred to as reports) from VigiBase that identified one or more anticancer treatments as suspect and included, or did not include, one or more pregnancy‐related reactions. Pregnancy‐related reports were defined as any report containing the following terms from MedDRA: Pregnancy, puerperium, and perinatal conditions (system organ classification–SOC), Fetal and neonatal investigations (high‐level group term–HLGT), Neonatal and perinatal conditions (HLGT), Neonatal respiratory disorders (HLGT), Exposures associated with pregnancy, delivery, and lactation (high‐level term–HLT), Fetal therapeutic procedures (HLT), Induced abortions (HLT), and Obstetric therapeutic procedures (HLT). Details are summarized in **Table** **S1**.

A report was considered suspect when one or more anticancer treatments were listed as “Suspect” or “Interacting.” Anticancer treatments were defined as those within the “antineoplastic” category, Anatomical Therapeutic Classification (ATC) group L01. Reports involving endocrine therapy (ATC group L02) alone were not considered.

### Data cleaning and exclusion criteria

We ensured that only reports mentioning pregnancy‐associated conditions/exposure were retained with terms primarily associated with pregnancy as a main SOC, HLGT, or HLT. We discarded reports with terms secondarily associated with pregnancy.

Reports were then analyzed to discard:

‐ Reports with no mention of a cancer diagnosis or with an antineoplastic treatment from the L01 ATC group prescribed for a non‐cancer indication (e.g., alemtuzumab for multiple sclerosis or methotrexate for rheumatoid arthritis).

‐ Reports with drug mapping problems or adverse event mapping problems (**Table** **S2**).

‐ Identification of duplicated reports using an information‐entropy‐based in‐house algorithm (cf. **Supplementary Methods**).

‐ Dyads, that is, maternal and fetal reports referring to the same case, were detected using an in‐house based algorithm and merged (cf. **Supplementary Methods**).

### Modality of exposure during pregnancy

The timing and mode of anticancer treatment exposure were determined using preferred terms noted in the reports (see **Table** **S3** for terms and exposure modalities). The types of exposure included: exposure before pregnancy, during pregnancy, via breast milk, via semen, and via skin. Reports involving individuals identified as male in non‐pediatric groups were classified as exposed via semen. Reports indicating exposure via skin or semen were excluded, as were those specifying exposure via breast milk or before pregnancy without clear mention of exposure during pregnancy. All other reports were classified as having anticancer treatment exposure during pregnancy.

### Definition of exposure groups

We considered the following treatments to be used for PrBC: paclitaxel, docetaxel, doxorubicin, epirubicin, cyclophosphamide, carboplatin, capecitabine, vinorelbine, fluorouracil, everolimus, pembrolizumab, trastuzumab, pertuzumab, trastuzumab emtansine, trastuzumab deruxtecan, tucatinib, neratinib, palbociclib, ribociclib, abemaciclib, sacituzumab govitecan, lapatinib, olaparib, talazoparib.

Any report from the study analysis with a mention of a treatment used in BC was qualified as a “BC treatment.” Each report within the BC treatment exposure group was individually analyzed. Reports with other anticancer treatments and no mention of a treatment used in BC were qualified as the “exposure to other anticancer” group.

### Definition of cases and non‐cases for maternal and fetal/newborn outcomes

In this disproportionality analysis, cases were defined as reports that mentioned maternal and/or fetal/newborn adverse events, categories based on MedDRA preferred terms in VigiBase. These cases represented 37 individual maternal–fetal adverse outcomes, which were grouped into five categories for this study: Congenital malformation, Neonatal complication, Pregnancy complication, Preterm birth, and Fetal death/stillbirth. Further details on the fetal toxicity subtypes analyzed are provided in **Table** **S4**. Some adverse outcomes were considered not clinically relevant and were excluded from the analysis (see **Table** **S5**).

For the analysis, cases were defined as reports from the study population that mentioned the specific adverse event. Non‐cases were all other reports from the study population that did not mention the adverse event.

### Statistical analysis

We conducted a case/non‐case study using disproportionality analysis to assess the association between an adverse outcome of interest and exposure to a drug. The reporting odds ratio (ROR) was calculated as the ratio of the odds of the adverse pregnancy or fetal/newborn outcome of interest following exposure to a BC treatment compared to exposure to other anticancer treatments. The primary analysis individually assessed the four pre‐specified agents (doxorubicin, epirubicin, paclitaxel, docetaxel), representing the two cornerstone cytotoxic classes (anthracyclines and taxanes) used in pregnancy‐associated BC and enabling within‐class comparisons; all other BC treatments were assessed as secondary analyses. We conducted an individual analysis of all reports involving the four pre‐specified agents, doxorubicin and epirubicin (anthracyclines) and paclitaxel and docetaxel (taxanes), to investigate drug‐specific signals within each pharmacologic class and enable within‐class comparisons. The ROR confidence interval calculation was adjusted for multiple testing based on the number of drugs investigated. Drugs investigated were every BC treatment provided with 20 or more occurrences of the molecule in the entire cohort.

A signal of disproportionate reporting was considered present if the lower bound of the ROR confidence interval (ROR025) exceeds one and the number of occurrences of cases is three or more. The study population was characterized using frequencies for qualitative variables and medians with interquartile ranges (IQR) for quantitative variables. Associations between categorical variables were tested using Fisher's exact test, and *P*‐values less than 0.05 were considered statistically significant.

### Sensibility and subgroup analysis

To assess the robustness of our results, we conducted sensitivity analyses on reports with BC identified (treatments used in BC with actual BC identified). We applied the same method used for reports involving BC treatments to those who had an identified BC. We also examined whether maternal–fetal complications are limited to first‐trimester (T1) exposure or if they occur following administration during the second or third trimester.

### Data protection

The data in the VigiBase database are anonymized, preventing access to any personal information about the patients or individuals who reported the cases. We complied with all relevant legislation, including but not limited to EU and national regulations on personal data protection, such as the Data Protection Directive 95/46/EC and Regulation (EC) No. 45/2001, where applicable. The study adhered to the Reporting of a Disproportionality Analysis for Drug Safety Signal Detection Using Individual Case Safety Reports in PharmacoVigilance (READUS‐PV^20^) checklist (**Table** **S6**). Strengthening the Reporting of Observational Studies in Epidemiology (STROBE) guidelines for case–control studies (**Table** **S7**).^21^ Study approval was granted by the institutional review board of Institut Curie (Comité de Revue Institutionnelle‐CRI Data).

## RESULTS

### Reports characteristics

We extracted 10,832 deduplicated reports and retained 3310 reports of pregnant individuals exposed to anticancer treatments for the final analysis (**Figure** **S1**) (BC treatment group, *n* = 1,789; other anticancer treatment group, *n* = 1521). Mean (SD) age was 27.4 (13.8) vs. 29.3 (11.7) years old in the BC treatment group vs. other anticancer treatment group. North America accounted for 46.3% of overall reports but 58.0% of BC treatment group reports, likely reflecting higher US reporting rates for breast cancer adverse events; this geographic over‐representation is acknowledged as a potential source of reporting bias (see Limitations; missing data by variable are reported in Supplemental **Table** **S12**). Within the BC treatment group with known cancer type, breast cancer (*n* = 908, 58%) was the most frequent type followed by lymphoma (*n* = 298, 19%) and sarcoma (*n* = 72, 5%). In the exposed to other anticancer treatment group, chronic myeloid leukemia (*n* = 727, 56%), acute leukemia (*n* = 156, 12%) and lymphoma (*n* = 54, 4%) were the most frequent cancer type. (**Table** **1**; **Figure** **S2**). Main molecules used in the BC treatment group (*n* = 1789) were cyclophosphamide (*n* = 976, 54.6%), doxorubicin (*n* = 919, 51.4%), fluorouracil (*n* = 412, 23.0%), paclitaxel (*n* = 295, 16.5%), trastuzumab (*n* = 291, 16.3%), epirubicin (*n* = 123, 6.9%), docetaxel (*n* = 112, 6.3%), and carboplatin (*n* = 119, 6.7%). BC treatments were used in combination in 1026 participants (57.4%), of whom 512 (49.9%) received two molecules, 416 (40.5%) received three molecules, and 98 (9.6%) received four or more (**Table** **S8**). In the other anticancer group (*n* = 1,521), reports mentioned mainly molecular targeted therapy including 630 imatinib (41.4%), 215 nilotinib (14.1%), 127 dasatinib (8.3%), cytotoxic therapy including 78 (5.1%) cytarabine, 59 (3.9%) daunorubicin, 55 (3.6%) cisplatin (full data available in **Table** **S9**).

**Table 1:** Characteristics of pharmacovigilance reports from pregnant individuals exposed to BC anticancer treatments vs. other anticancer treatments in VigiBase (query: January 2024, most recent available reports: 2023)

	Overall	PrBC anticancer drug	Other anticancer drugs	*p*
	*n* (%)	*n* (%)	*n* (%)	
Total number of reports	3310	1789 (54)	1521 (46)	
age—Mean in years (SD)	28.4 (12.7)	27.4 (13.8)	29.3 (11.7)	0.004
Region				
North America	1533 (46.3)	1037 (58.0)	496 (32.6)	< 0.001
Europe	882 (26.7)	493 (27.6)	389 (25.6)	
Other regions	667 (20.2)	177 (9.9)	490 (32.2)	
Eastern Asia	227 (6.9)	82 (4.6)	145 (9.5)	
Notifier type				
Physician or pharmacist	1631 (52.6)	759 (45.8)	872 (60.6)	< 0.001
Other health professional	1160 (37.4)	788 (47.5)	372 (25.9)	
Consumer or non‐health professional	307 (9.9)	112 (6.8)	195 (13.6)	
Number of notifiers				
1	2779 (89.7)	1603 (96.6)	1176 (81.7)	< 0.001
2+	319 (10.3)	56 (3.4)	263 (18.3)	
Year of first report				
2009 or before	294 (8.9)	143 (8.0)	151 (9.9)	< 0.001
2010–2014	759 (22.9)	526 (29.4)	233 (15.3)	
2015–2019	1330 (40.2)	635 (35.5)	695 (45.7)	
2020–2023	927 (28.0)	485 (27.1)	442 (29.1)	
Number of suspect/interacting drugs				
1	1449 (43.8)	433 (24.2)	1016 (66.8)	< 0.001
2	572 (17.3)	327 (18.3)	245 (16.1)	
3+	1288 (38.9)	1029 (57.5)	259 (17.0)	
Clinical trial				
Routine care	3256 (98.4)	1758 (98.3)	1498 (98.6)	0.608
Clinical trial	53 (1.6)	31 (1.7)	22 (1.4)	
Cancer type*				
Breast	908 (32.0)	908 (58.1)	0 (0.0)	< 0.001
Chronic myeloid leukemia	721 (25.4)	0 (0.0)	721 (56.6)	
Lymphoma	360 (12.7)	298 (19.1)	62 (4.9)	
Solid tumor other or NOS	253 (8.9)	98 (6.3)	155 (12.2)	
Acute leukemia	208 (7.3)	54 (3.5)	154 (12.1)	
Sarcoma	97 (3.4)	72 (4.6)	25 (2.0)	
Ovarian peritoneal gestational & germline	75 (2.6)	47 (3.0)	28 (2.2)	
Cervix vagina & vulva	63 (2.2)	50 (3.2)	13 (1.0)	
Hematological other or NOS	52 (1.8)	13 (0.8)	39 (3.1)	
NSCLC	51 (1.8)	12 (0.8)	39 (3.1)	
Melanoma	48 (1.7)	10 (0.6)	38 (3.0)	

*Note:* Quantitative values are represented as mean and standard deviation. When the total of patients in the overall cohort was < 50, the organ affected was included in solid tumor other or NOS: it concerned pancreas, lung NOS, gallbladder & bile duct, head & neck, endometrium, other tumors, SCLC, thymus, renal, mesothelioma, endocrine (not thyroid), brain & nervous system, colorectal & intestine, leukemia other or NOS, myeloma, gastroesophageal, skin (not melanoma), liver, digestive other or NOS. Missing data: (i) For BC treatment group: age (years), *n* = 1,134; notifier type, *n* = 130; number of notifiers, *n* = 130; Number of cancers, *n* = 255. (ii) For other anticancer treatment: age (years), *n* = 697; country group, *n* = 1; notifier type, *n* = 82; number of notifiers, *n* = 82; number of Suspect/Interacting drugs, *n* = 1; Clinical trial, *n* = 1; Number of cancers, *n* = 268.

Abbreviations: BC, Breast Cancer; NOS, Not Otherwise Specified; NSCLC, non‐small cell lung cancer; USA, United States of America.

* Patients could have two cancers simultaneously.

### Pregnancy and/or fetal or newborn outcomes

Pregnancy or fetal/newborn adverse outcomes were found in 998 reports (55.8%) in the BC treatment group and 470 reports (30.9%) in the other anticancer treatment group. There were across all reports 1,595 total fetal toxicity reported in the BC treatment group and 783 in the other anticancer group with ROR = 2.8 [95% CI 2.4–3.2]. The five complications most frequently reported in the group receiving BC treatment were preterm birth (*n* = 566, 35.5%), intrauterine growth restriction (*n* = 241, 15.1%), oligohydramnios (*n* = 124, 7.8%), neonatal respiratory disorder (*n* = 111, 7.0%), and fetal death‐stillbirth (*n* = 80, 5.0%), **Table** **S10**.

### Primary analysis: Drug‐specific signals for Anthracyclines (doxorubicin, Epirubicin) and Taxanes (paclitaxel, docetaxel)

**Figure 1:**
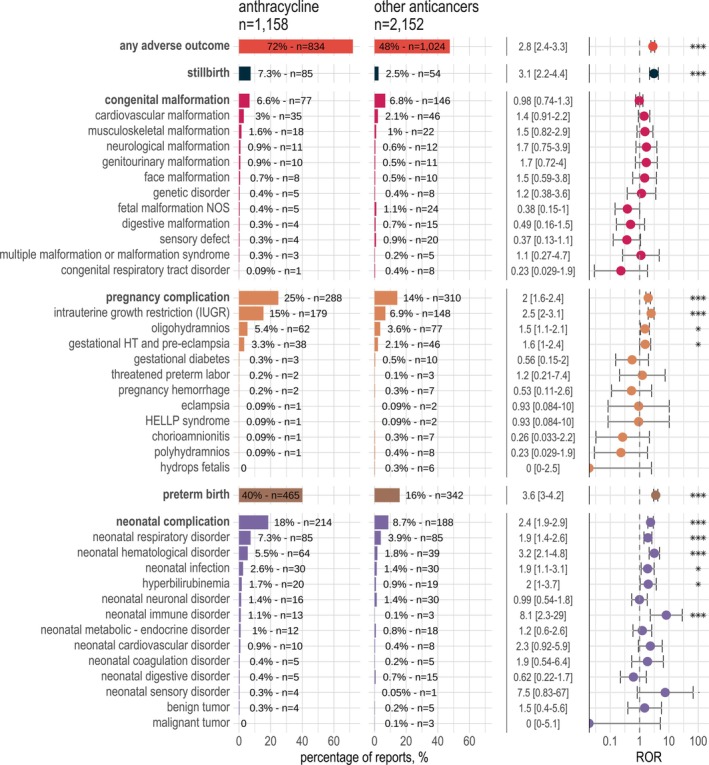
Description and reporting odds ratio (ROR) of pregnancy and fetal/newborn adverse outcomes with exposure to anthracycline compared to exposure to other anticancer drugs. Bars represent the percentage of each outcome divided by the total number of reports. Counts are annotated. RORs are displayed as logarithmic data for the purpose of data visualization. Among the 45 pre‐specified maternal–fetal adverse outcome types, we represented (**Table** **S41**) toxicities for which at least one case was found in the TKI‐exposed group. *P*‐values: *P‐value ≤ 0.05, **P‐value ≤ 0.01, ***P‐value ≤ 0.001. When the N observed was one, the result was not considered significant. Abbreviations: HT: Hypertension; NOS: Not Otherwise Specified; Of note, for epirubicin and musculoskeletal malformation: ROR025 was originally superior to 1, but was not maintained when manual removal of an identified duplicated case and resulted in non‐significant signal.

Compared to anticancer treatment group, overall anthracycline exposure (*n* = 1,158) was associated with several neonatal and pregnancy complications, preterm birth and stillbirth (**Figure** **1**). Specifically, doxorubicin (*n* = 919) exposure was associated with neonatal immune deficiency (*N*_obs_ = 13, ROR = 11.0 95% CI 3.2–40), neonatal hematological disorder (*N*_obs_ = 40, ROR = 1.7 95% CI 1.1–2.5 from which 33 cases had cyclophosphamide administered in combination), neonatal respiratory disorder (*N*_obs_ = 63, ROR = 1.6 95% CI 1.1–2.2); with hypertension/pre‐eclampsia (*N*_obs_ = 35, ROR = 1.9 95% CI 1.2–2.9), IUGR (*N*_obs_ = 141, ROR = 2.1 95% CI 1.7–2.7) and stillbirth (*N*_obs_ = 65, ROR = 2.4 95% CI 1.7–3.4). Epirubicin (*n* = 123) was associated with specific outcomes: face malformations (*N*_obs_ = 4, ROR = 7.6 95% CI 2.5–23.5), genitourinary malformations (*N*_obs_ = 4, ROR = 6.3 95% CI 2.1–18.9) and oligohydramnios (*N*_obs_ = 12, ROR = 2.6, 95% CI 1.4–4.8) (**Figure** **3**).

**Figure 2:**
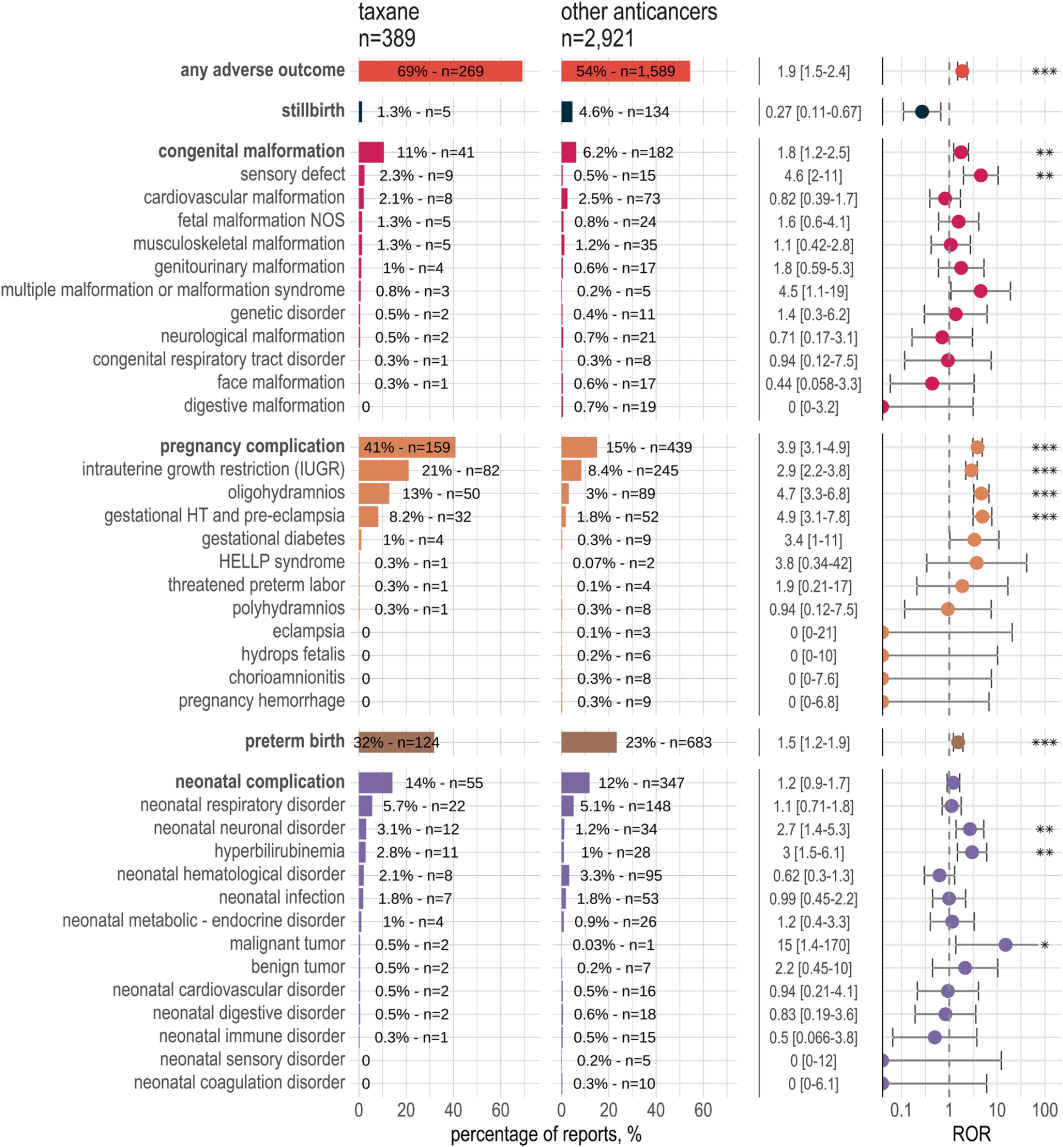
Description and reporting odds ratio (ROR) of pregnancy and fetal/newborn adverse outcomes with exposure to taxane compared to exposure to other anticancer drugs. Bars represent the percentage of each outcome divided by the total number of reports. Counts are annotated. RORs are displayed as logarithmic data for the purpose of data visualization. Among the 45 pre‐specified maternal–fetal adverse outcomes types, we represented (**Table** **S4**) toxicities for which at least one case was found in the TKI‐exposed group. *P*‐values: *P‐value ≤ 0.05, **P‐value ≤ 0.01, ***P‐value ≤ 0.001. When the N observed was one, the result was not considered significant. Abbreviations: HT: Hypertension; NOS: Not Otherwise Specified.

**Figure 3:**
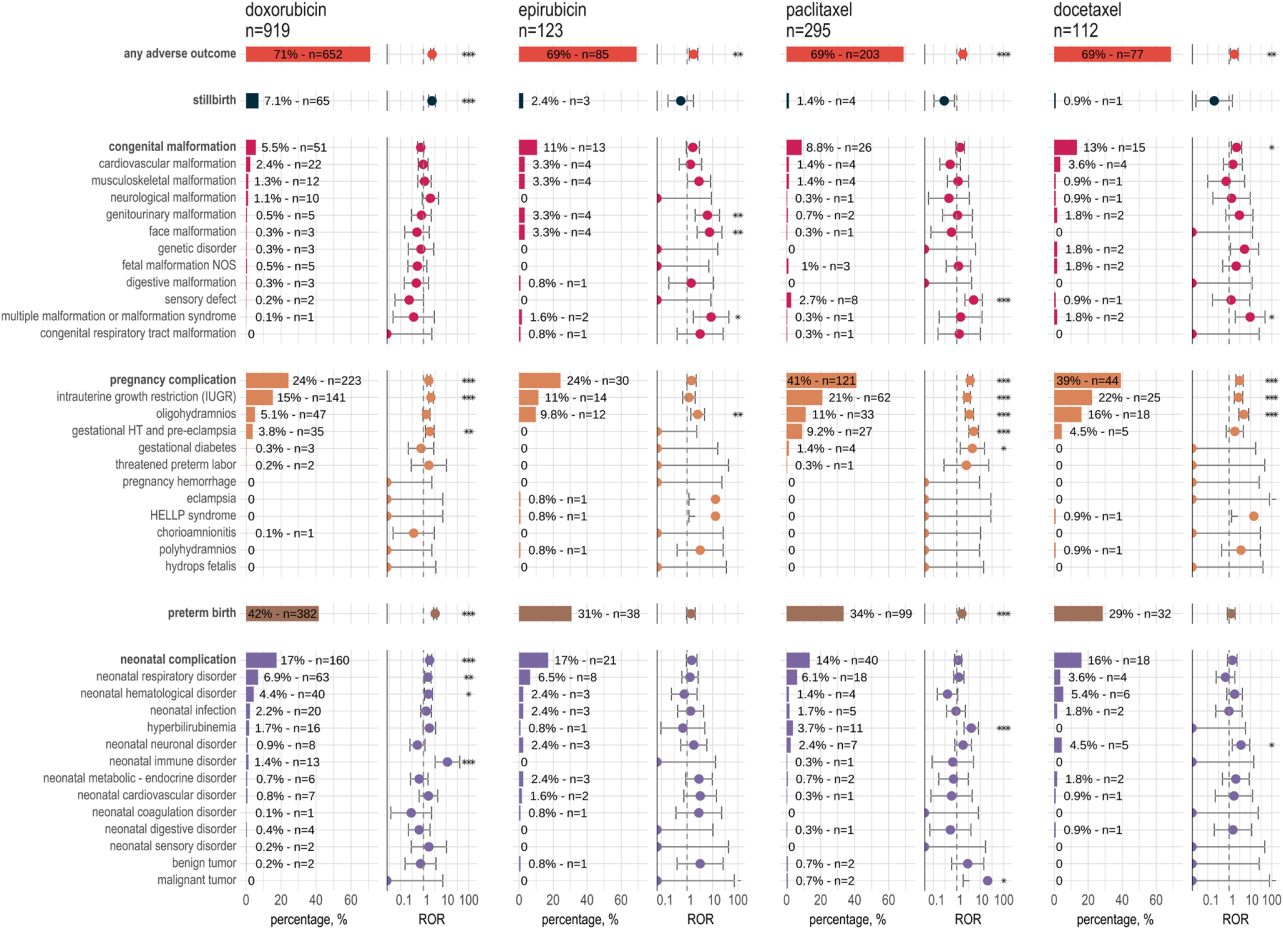
Description and reporting odds ratio (ROR) of pregnancy and fetal/newborn adverse outcomes with exposure to all four main molecules used in BC: doxorubicin, epirubicin, paclitaxel, docetaxel individually. Bars represent the percentage of each outcome divided by the total number of reports. Counts are annotated. RORs are displayed as logarithmic data for the purpose of data visualization. *P*‐values: *P‐value ≤ 0.05, **P‐value ≤ 0.01, ***P‐value ≤ 0.001. When the *N* observed was one, the result was not considered significant.

Overall taxane exposure (n = 389) was significantly associated with congenital malformation, hyperbilirubinemia, neonatal neuronal disorder, pregnancy complications, in particular placental and vascular complications, and preterm birth (**Figure** **2**). Among taxanes, both paclitaxel and docetaxel displayed overlapping vascular‐placental toxicities: IUGR (*N*_obs_ = 62, ROR = 2.8, 95% CI 2.0–3.8 for paclitaxel and *N*_obs_ = 25, ROR = 2.8, 95% CI 1.7–4.4 for docetaxel) and oligohydramnios (*N*_obs_ = 33, ROR = 3.5, 95% CI 2.3–5.2 for paclitaxel and *N*_obs_ = 18, ROR = 4.9, 95% CI 2.8–8.3 for docetaxel) (**Figure** **3**). From the 50 cases reported of oligohydramnios with taxanes, 36 (72%) had concomitant anti‐HER2 therapies.

Paclitaxel was associated with sensory defects (*N*_obs_ = 8, ROR = 5.2, 95% CI 2.2–12.3), neonatal hyperbilirubinemia (*N*_obs_ = 11, ROR = 4.1, 95% CI 2.0–8.4), gestational diabetes (*N*_obs_ = 4, ROR = 4.6, 95% CI 1.4–15), hypertension/pre‐eclampsia (*N*_obs_ = 27, ROR = 5.2, 95% CI 3.3–8.4) and preterm birth (*N*_obs_ = 99, ROR = 1.6, 95% CI 1.3–2.1). Docetaxel exposure (*n* = 112) was associated with neonatal neuronal disorders (*N*_obs_ = 5, ROR = 3.6 95% CI 1.4–9.3).

**Figure 4:**
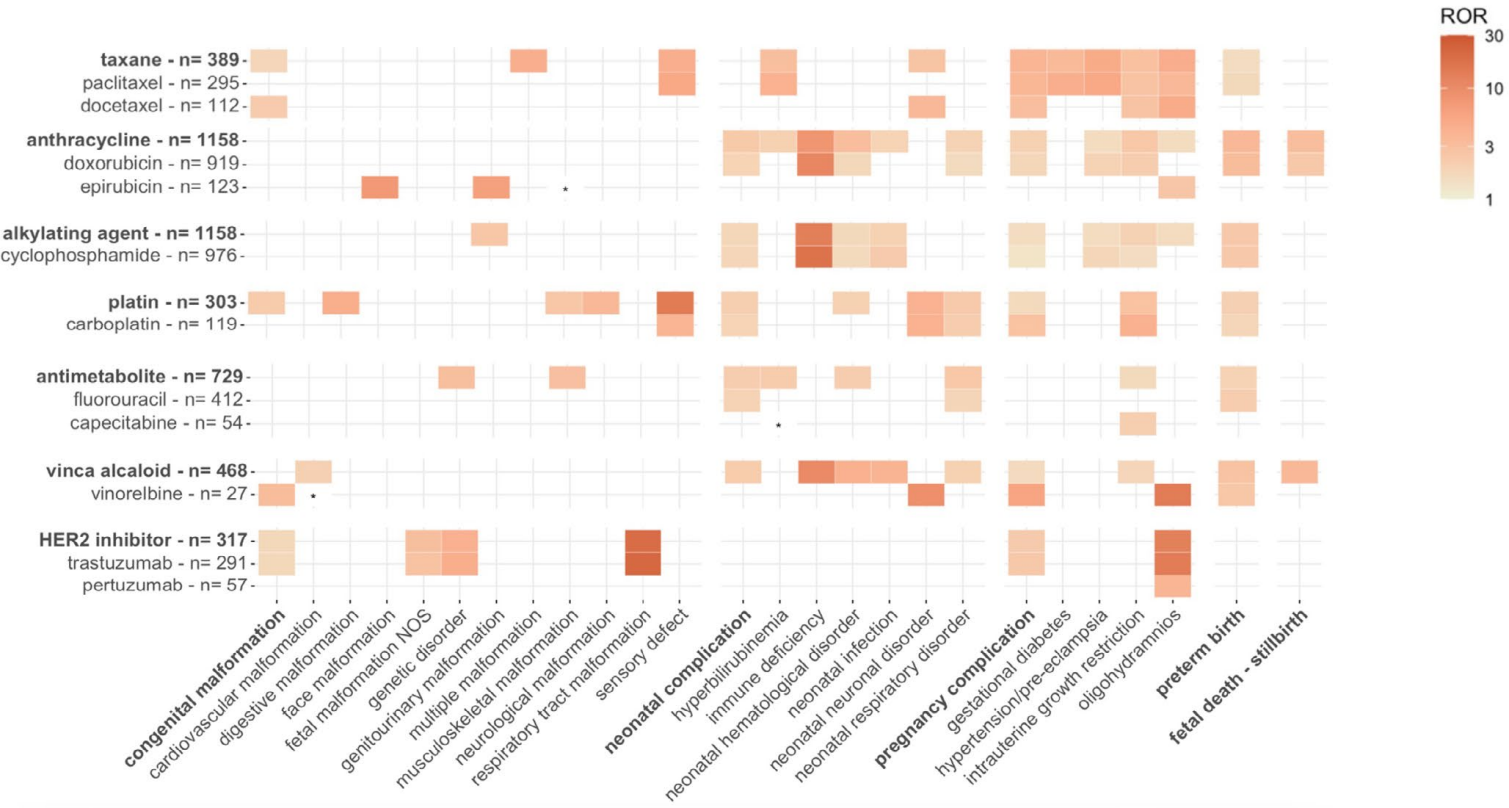
Heatmap presenting reporting odds ratio (ROR) of molecules used in pregnancy‐associated breast cancer, restrained to those with ROR025 > 1 and with at least two reported cases of any fetal toxicity type. Fetal toxicity types with no overreporting have been removed from the heatmap to improve readability: benign tumor, malignant tumor, neonatal cardiovascular disorder, neonatal coagulation disorder, neonatal digestive disorder, neonatal metabolic–endocrine disorder, neonatal sensory disorder, chorioamnionitis, eclampsia, HELLP syndrome, hydrops fetalis, polyhydramnios, pregnancy hemorrhage, threatened preterm labor. Molecules with less than 20 occurrences are not displayed. Full data are shown in **Table** **S11** and **Figure** **S2**. *ROR025 was originally superior to 1, but was not maintained when manual removal of duplicated cases was resulted in non‐significant signal. [Correction added on 2 August 2026, after first online publication: Figure 4 was duplicated in the original publication and has been replaced with the correct figure in this version.]

Most cases of cyclophosphamide were associated with anthracycline (835/976, 85.5%) and cyclophosphamide mirrored anthracycline toxicities with neonatal immune deficiency (Nobs = 14, ROR = 17.0 95% CI 3.8–75), infections (Nobs = 29, ROR = 2.3 95% CI 1.4–3.8), and hematological disorders (Nobs = 41, ROR = 1.6 95% CI 1.1–2.4), hypertension/pre‐eclampsia (Nobs = 35, ROR = 1.7, 95% CI 1.1–2.7), IUGR (Nobs = 123, ROR = 1.5 95% CI 1.2–1.9), and preterm birth (Nobs = 355, ROR = 2.4, 95% CI 2.0–2.8). No significant disproportionate reporting was identified for trastuzumab‐emtansine (*n* = 21), pembrolizumab (*n* = 36), or lapatinib (*n* = 13). No reports were available for talazoparib. Within the main analysis, molecules with less than 20 occurrences were not added to the main analysis (*n* = 9 for palbociclib; *n* = 4 for everolimus; *n* = 3 for ribociclib; *n* = 2 for sacituzumab govitecan, trastuzumab deruxtecan, tucatinib, olaparib; *n* = 1 for neratinib, and abemaciclib). A heatmap of reporting odds ratios for other molecules is available in **Figure** **4** and full data with all molecules are shown in **Table** **S11** and **Figure** **S3**.

### Impact of trimester exposure

**Figure 5:**
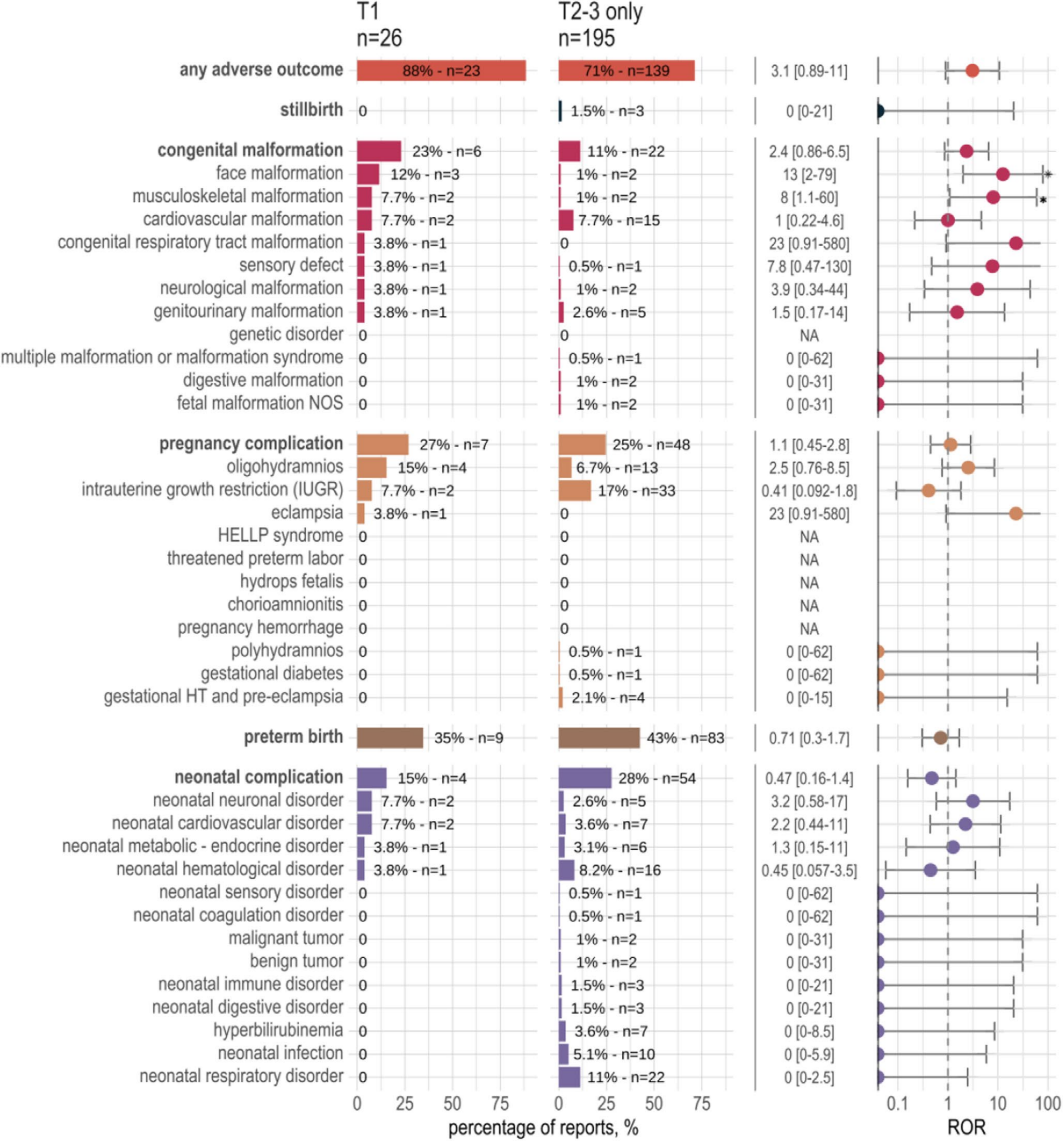
Reporting Odds Ratios (ROR) for neonatal and pregnancy complications in all reports with known trimester exposure (*n* = 221) involving at least one of the four commonly used BC drugs (paclitaxel, docetaxel, doxorubicin, or epirubicin), comparing first‐trimester (T1) exposure with exposure after the first trimester (T2/T3).

We individually analyzed all 1,336 reports mentioning at least one of the four commonly used treatments in BC: paclitaxel, docetaxel, doxorubicin, and epirubicin. Trimester administration timing was available within 221 reports: 195 reports exposition after T1 and 26 during T1 (13 epirubicin, 7 doxorubicin, 3 docetaxel, 2 paclitaxel, 1 mentioning both paclitaxel, and doxorubicin). Administration during the first trimester was associated with a significantly higher number of neonatal and pregnancy complications compared to exposure in later trimesters. Main complications that were reported with T1 (compared to T2/T3) were some congenital malformations: facial malformation (ROR = 13, 95% CI 2–79) and musculoskeletal malformation (ROR = 8, 95% CI 1.1–60) (**Figure** **5**).

### Sensitivity and subgroup analysis

In the sensitivity analysis restricted to the subpopulation with confirmed BC diagnosis (*n* = 908), main signals were preserved (**Figure** **S4**). When the comparator was restricted to cytotoxic‐only agents (*n* = 262), key signals remained directionally consistent: paclitaxel retained associations with hypertension/pre‐eclampsia (ROR = 5.3 [2.6–10.7]), IUGR (ROR = 1.9 [1.2–2.9]) and oligohydramnios (ROR = 2.2 [1.2–3.8]); epirubicin retained congenital malformation signals; and cyclophosphamide showed immune deficiency and preterm birth signals (**Figure** **S5**). When anthracycline exposure was restricted to reports without cyclophosphamide co‐administration, doxorubicin retained IUGR, preterm birth, stillbirth, and neonatal respiratory disorder signals, while the neonatal hematological disorder signal was absent, supporting a cyclophosphamide‐driven mechanism (**Figure** **S6**).

## DISCUSSION

In this VigiBase pharmacovigilance study, we analyzed over 3,000 reports of anticancer drug exposure during pregnancy to identify signals of disproportionate reporting of maternal and fetal risks associated with BC treatments. Our findings are consistent with, and extend, prior observations that antineoplastic agents during pregnancy are associated with specific adverse pregnancy and neonatal outcomes; they should be interpreted as hypothesis‐generating signals requiring confirmation in prospective registries.

First, we observed a high proportion of maternal–fetal adverse outcomes in the BC treatment group (55.8%) compared to the other anticancer treatment group (30.9%). These differences likely reflect not only the specific pharmacologic toxicity profiles of BC treatments but also the intensity and combination of agents used, as over half of the exposed patients to BC treatments received multidrug regimens often combined with cytotoxic regimens. However, our analysis revealed distinct safety patterns associated with individual chemotherapeutic agents commonly used in BC. Anthracyclines and doxorubicin specifically were associated with increased risks of multiple neonatal complications (i.e., hematological disorder, immune deficiency, respiratory disorder, infections), pregnancy complications (hypertension/pre‐eclampsia, IUGR), and stillbirth in accordance with some existing literature^13^, ^22^, ^23^ without overreporting of cardiovascular malformation as previously suggested.^24^ Epirubicin was linked to specific facial and genitourinary malformations, representing a previously unreported safety signal, with no association with the trimester of exposure. Previous specific data on epirubicin risk of use in pregnant patients are scarce.^24^, ^25^ Epirubicin is the 4′‐epimer stereoisomer of doxorubicin and they therefore have a very close structure. BCRP is a protein expressed as a gatekeeper in the blood‐placental barrier. Over‐expression of BCRP by tumor cells confers resistance to both epirubicin and doxorubicin^26^ and they are both expected to cross very little of the blood‐placental barrier. Their pharmacokinetic profiles differ mainly due to glucuronic acid conjugates of epirubicin, possibly leading to fewer exposures to doxorubicinol.^27^ Their spectrum of toxicity is also known to differ slightly and epirubicin is known for less myelosuppression and lesser cardiotoxicity.^27^, ^28^, ^29^ These differences could explain a slightly different safety profile with maternofetal exposure, although a more important rate of prescription within the first trimester compared to doxorubicin might be possible.

Among the 389 reports involving taxane exposure during pregnancy, several adverse events were reported, including sensory defects, hyperbilirubinemia, neonatal neurologic disorders, gestational diabetes, hypertension/pre‐eclampsia, intrauterine growth restriction, and oligohydramnios. Although some of these outcomes are clinically serious, each occurred infrequently, and the overall number of exposed pregnancies remains limited. Overall, these findings suggest that taxane use during pregnancy may be associated with a relatively favorable safety profile, likely due to low placental transfer and the protective role of P‐glycoprotein.^14^, ^30^, ^31^ Most of the reported adverse events were primarily observed with paclitaxel, likely reflecting its more frequent use. Our findings are consistent with case series,^32^, ^33^, ^34^ reporting associations with hepatic complications (e.g., hyperbilirubinemia) and fetal sensory defects. Importantly, All eight reports of sensory defects in paclitaxel‐exposed pregnancies co‐reported platinum exposure (cisplatin: *n* = 5; carboplatin: *n* = 3), and no cases occurred without platinum co‐administration. For the five cisplatin‐exposed cases, platinum‐induced ototoxicity is the most parsimonious explanation, given cisplatin's well‐documented cochleotoxicity. For the three carboplatin‐exposed cases, the interpretation is more nuanced: carboplatin ototoxicity in children receiving high‐dose protocols is established, but its fetal ototoxic potential remains largely unknown. These three cases may therefore represent a novel pharmacovigilance signal of in utero carboplatin‐associated auditory toxicity, distinct from the cisplatin mechanism. This distinction warrants dedicated investigation in future prospective registries collecting audiological outcomes in children born to mothers exposed to carboplatin‐containing regimens. Docetaxel showed specific disproportionate reporting of neuronal disorders partly described in the US National Toxicology Program's clinical outcome tracking. Oligohydramnios has previously been attributed to anti‐HER2 therapies, and the apparent taxane signal was mainly driven by anti‐HER2 co‐administration: 36 of 50 oligohydramnios cases (72%) co‐reported an anti‐HER2 therapy, a drug class with established oligohydramnios risk (annotated † in **Figures** **2**, **3**, **4**).

As previously described, we found that most complications seem to be more frequent when the molecule is administered during T1. Our findings reinforce the long‐standing clinical practice of avoiding chemotherapy during the first trimester, where the balance between maternal treatment needs and fetal safety is most precarious.^35^

Considered relatively safe,^12^, ^36^ our data are reassuring with cyclophosphamide exposure: we found an overreporting of hematologic and immune complications, non‐specific pregnancy‐related complications such as IUGR and preterm birth as previously described.^37^ Notably, anthracyclines and cyclophosphamide are frequently co‐administered in clinical practice. Because of this overlap, it is difficult to fully identify the individual contributions of each treatment to the observed adverse outcomes: some of the significant ROR observed in this study may reflect combined effects.

These findings are consistent with, and provide large‐scale pharmacovigilance support for, current guidelines, and may inform future clinical risk assessment strategies. Two pre‐specified sensitivity analyses were conducted to address the methodological concerns raised during peer review. First, restricting the comparator group to cytotoxic‐only agents (*n* = 262; excluding the TKI‐dominated primary comparator) confirmed key drug‐specific signals: paclitaxel retained signals for hyperbilirubinemia (ROR = 3.4 [1.2–9.2]), hypertension/pre‐eclampsia (ROR = 5.3 [2.6–10.7]), IUGR (ROR = 1.9 [1.2–2.9]), and oligohydramnios (ROR = 2.2 [1.2–3.8]); epirubicin retained congenital malformation signals (**Figure** **S5**). The sensory defect signal was also retained (ROR = 3.7 [1.1–12.4]); however, this reflects the same eight platinum‐co‐exposed cases identified in the primary analysis rather than independent evidence of a paclitaxel‐specific effect, and should be interpreted with the same caution. Second, restricting to anthracycline exposure without cyclophosphamide confirmed that IUGR, preterm birth, fetal death, and neonatal respiratory disorder signals are attributable to doxorubicin independently, while the neonatal hematological disorder signal was absent in the monotherapy subgroup, supporting a cyclophosphamide‐driven mechanism (**Figure** **S6**). First‐trimester administration carries high teratogenic risk and should be avoided when possible, in line with current guidelines. Regimens associated with higher rates of neonatal adverse outcomes—particularly anthracycline–cyclophosphamide combinations, which increase neonatal myelosuppression risk—could be scheduled as far as possible from delivery. In contrast, taxane‐based regimens are preferable in the peri‐delivery period, though taxanes may carry more pregnancy‐specific vascular‐placental complications and are preferably administered later during pregnancy.

Several limitations of our study should be acknowledged. Underreporting and reporting bias are intrinsic to pharmacovigilance data. Adverse events are more likely to be reported when severe, preventing a precise estimation of risks. Incomplete clinical data, including dose, gestational age at exposure, and pregnancy outcomes, also limit causality assessment. Potential confounding by indication or co‐medication could not be fully accounted for. In particular, the primary comparator group is dominated by TKI‐treated patients (imatinib 41.4%, nilotinib 14.1%, dasatinib 8.3%), creating an imperfect reference for cytotoxic BC regimens. Additionally, dedicated CML pregnancy surveillance programs may have led to more complete reporting in the imatinib‐predominant comparator group, potentially leading to conservative (underestimated) RORs for BC treatments. However, a pre‐specified sensitivity analysis restricting the comparator to cytotoxic‐only agents confirmed the directionality of key signals (**Figure** **S5**); this analysis is itself underpowered for several endpoints, and the absence of a signal does not constitute evidence of absence of an effect. The near‐universal co‐administration of cyclophosphamide with anthracyclines (85.5%) limits individual drug attribution; a monotherapy‐restricted sensitivity analysis (**Figure** **S6**) partially addressed this limitation. They could also reinforce previously suspected signals or better describe the previously known first‐trimester chemotherapy‐induced teratogenicity.

Future prospective registries and cohort studies are needed to refine trimester‐specific and dose‐dependent risk estimates with long‐term outcomes of children exposed in utero to these agents. Lack of long‐term follow‐up data on neurodevelopmental outcomes, which may be particularly relevant for some agents. Emerging therapies (e.g., CDK4/6 inhibitors, PARP inhibitors, antibody‐drug conjugates) should be closely monitored as their use in younger women increases. Close fetal and post‐natal monitoring should be assessed depending on fetal toxicity type of each agent.

## CONCLUSION

This large‐scale pharmacovigilance study identifies signals of disproportionate reporting of adverse pregnancy and neonatal outcomes with BC treatments, with distinct drug‐ and trimester‐specific patterns. Two pre‐specified sensitivity analyses confirmed the robustness of key signals while clarifying that the neonatal hematological disorder signal associated with anthracyclines is primarily driven by cyclophosphamide co‐administration. These hypothesis‐generating findings require confirmation in prospective registries but could ultimately inform individualized treatment planning and targeted surveillance strategies to minimize fetal risk while effectively managing maternal BC.

## FUNDING

PG was funded by the academic program “Contrats ED: Programme blanc Institut Curie PSL” during this study. The Institut Curie RT2L research group (PG, ASH, FJ, ED, RK, FC, EL, FR) was supported by the academic program “SHS INCa” and is part of a research project on young women funded by Monoprix*. This work benefitted from a France 2030 Grant for the Women's Cancers Institute, referenced ANR‐23‐IAHU‐0006 (RK, FC, ASH).

## CONFLICT OF INTEREST

Paul Gougis reports consulting fees from BMS and Johnson & Johnson, an academic grant from Sanofi and travel accommodation by Eisai and Bayer. All other authors declared no competing interests for this work.

## AUTHOR CONTRIBUTIONS

R.K., P.G., F.J., F.C., B.P., L.S., and A.S.H. wrote the manuscript. R.K., P.G., F.R., and F.J. designed the research. R.K., P.G., and L.C. performed the research. R.K., E.D., P.G., K.B., B.L.V. and M.A.B. analyzed the data.

## Supporting information


Data S1.

## Data Availability

Publicly available datasets were analyzed in this study. The data can be obtained from: https://www.vigiaccess.org. This study has not been published or presented elsewhere. The information does not represent the opinion of the Uppsala Medical Center or the World Health Organization.
