# Supplementary material for: Maternal and Neonatal Complications Associated with Breast Cancer Systemic Treatments—A VigiBase Disproportionality Analysis Study
Source: Clin Pharmacol Ther. 2026 Jul 29:10.1002/cpt.70404. Online ahead of print. doi: 10.1002/cpt.70404 (PMC13417350; doi:10.1002/cpt.70404)
Supplement: Supplementary file 1 — Data S1. [file CPT-9999-0-s001.docx]

# Supplemental Materials

[Supplementary Figures 3](#_Toc229694543)

[Figure S1. Study flow-chart 3](#_Toc229694544)

[Figure S2. Characteristics of reports in study population. 4](#_Toc229694545)

[Figure S3. Heatmap presenting reporting odds ratio (ROR) of molecules used in breast cancer, with ROR025 > 1 and with at least one reported case of any fetal toxicity type 5](#_Toc229694546)

[Figure S4. Sensitivity analysis in Breast Cancer only. 6](#_Toc229694547)

[Figure S5. Sensitivity analysis with cytotoxic-only comparator group. 7](#_Toc229694548)

[Figure S6. Anthracycline monotherapy sensitivity analysis. 8](#_Toc229694549)

[Supplementary Tables 9](#_Toc229694550)

[Table S1. Detail of VigiBase query. 9](#_Toc229694551)

[Table S2. Terms corrected. 10](#_Toc229694552)

[Table S3. MedDRA Preferred Terms used to qualify reports’ exposure type. 11](#_Toc229694553)

[Table S4. 37 individual materno-fetal adverse outcomes explored 12](#_Toc229694554)

[Table S5. Terms deemed not clinically significant. 13](#_Toc229694555)

[Table S6. The Reporting of a Disproportionality Analysis for Drug Safety Signal Detection Using Individual Case Safety Reports in PharmacoVigilance (READUS‑PV) checklist 14](#_Toc229694556)

[Table S7. The Strengthening the Reporting of Observational Studies in Epidemiology (STROBE) checklist: guidelines for reporting observational studies. 26](#_Toc229694557)

[Table S8. Number of reports and type of anticancer involved in the BC treatments exposure group (n = 1,789), with number of molecules received by patient 31](#_Toc229694558)

[Table S9. Number of reports and type of molecule involved in the other anticancer treatment group n = 1,521) 32](#_Toc229694559)

[Table S10. Description of all pregnancy and fetal/newborn adverse outcomes among patients who received molecules used in BC treatments exposure group and other anticancer treatment group 33](#_Toc229694560)

[Table S11: Reporting Odds Ratios (ROR) of Fetal Toxicity by Drug and Adverse Drug Reaction (ADR) for all molecules or classes and all ADR 35](#_Toc229694561)

[Table S12. Missing data per variable in Table 1 (N total = 3,310 reports). 43](#_Toc229694562)

[Supplemental Methods 44](#_Toc229694563)

[Identification of reports addressing mother vs fetal/newborn 44](#_Toc229694564)

[Position of the problem 44](#_Toc229694565)

[Scoring system for each report 44](#_Toc229694566)

[Final attribution 45](#_Toc229694567)

[Deduplication algorithm 45](#_Toc229694568)

[Problem position 45](#_Toc229694569)

[Initial selection 46](#_Toc229694570)

[Fusion of duplicates 48](#_Toc229694571)

[Fusion of dyads 49](#_Toc229694572)

[Position of the problem 49](#_Toc229694573)

[Inclusion criteria 49](#_Toc229694574)

[Exclusion criteria 49](#_Toc229694575)

# Supplementary Figures

## Figure S1. Study flow-chart

All reports were extracted from VigiBase using Vigilyze. Details of data extraction are provided in TableS1. Terms associated secondarily to this request were not specific to pregnancy and were discarded. Reports also mentioned a suspect or interacting drug from the anatomical and therapeutic classification L ATC L01 class (antineoplastic drugs), which could have been prescribed for a cancer or a non-cancer indication.

“Reports” here refers to “Individual Case Safety Reports” retrieved from VigiBase.

*MedDRA terms queried for “Reaction” were: Pregnancy, puerperium and perinatal conditions (SOC), Fetal and neonatal investigations (HLGT), Neonatal and perinatal conditions (HLGT), Neonatal respiratory disorders (HLGT), Exposures associated with pregnancy, delivery and lactation (HLT), Fetal therapeutic procedures (HLT), Induced abortions (HLT), Obstetric therapeutic procedures (HLT).

Abbreviations: ATC: Anatomical Therapeutic Chemical classification; MedDRA: Medical Dictionary for Regulatory Activities; BC: Breast Cancer


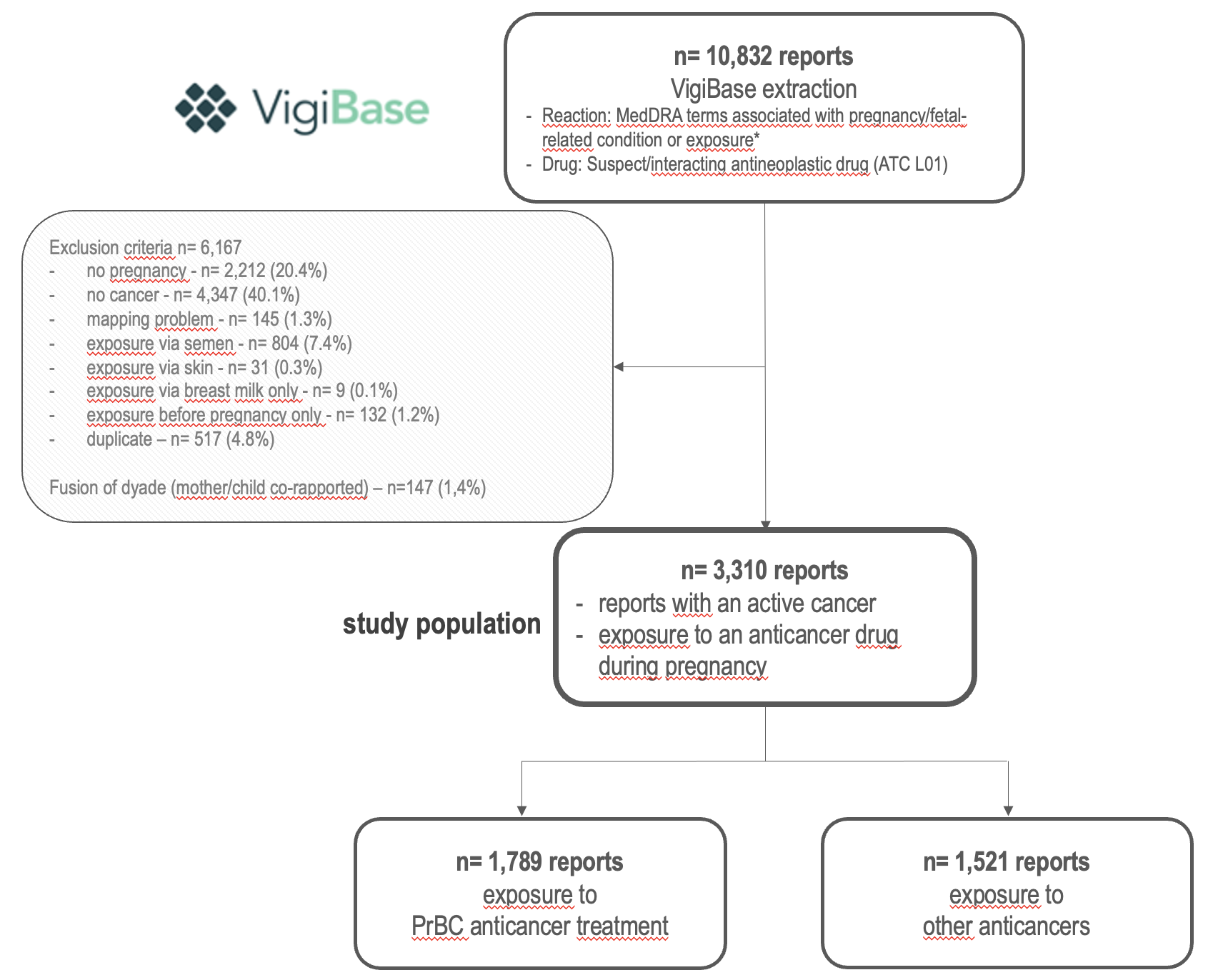


## Figure S2. Characteristics of reports in study population.

Characteristics of reports in pregnant population exposed to breast cancer treatments compared to exposure to other anticancer treatments. Panel A represent the age at diagnosis, panel B the year of report and panel C the cancer type identified within report. Two or more cancers could be diagnosed in a single report.

C : If total number of reports obtained by addition of both groups was <50, the organ affected was included in “solid tumor other or NOS” category. It concerned: pancreas, lung NOS, gallbladder & bile duct, head & neck, endometrium, other tumors, SCLC, thymus, renal, mesothelioma, endocrine (not thyroid), brain & nervous system, colorectal & intestine, leukemia other or NOS, myeloma, gastroesophageal, skin (not melanoma), liver, digestive other or NOS.

**
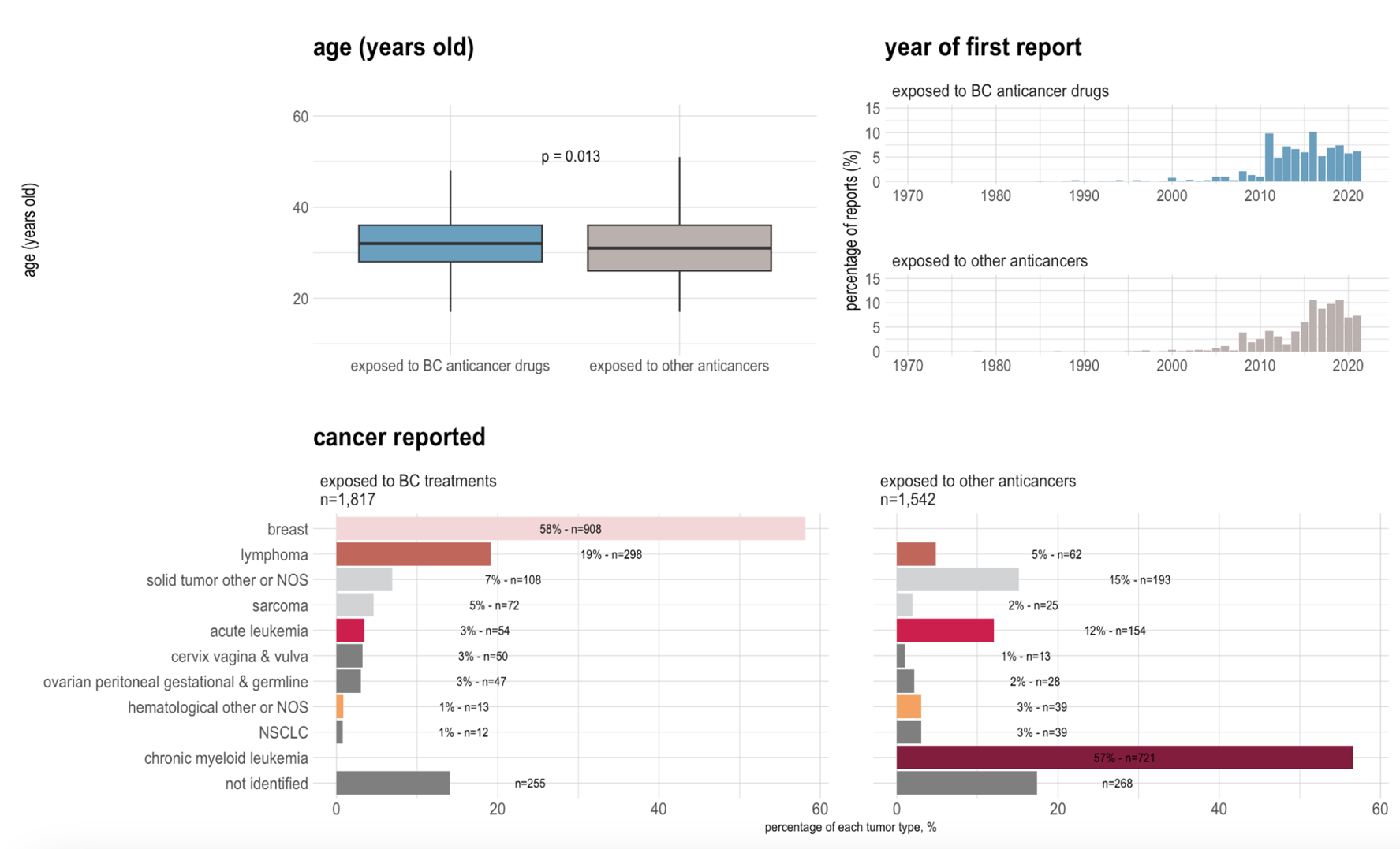
**

##

## Figure S3. Heatmap presenting reporting odds ratio (ROR) of molecules used in breast cancer, with ROR025 > 1 and with at least one reported case of any fetal toxicity type

For clarity, ADRs with no molecule having ROR025 ≥ 1 have been omitted.

*ROR025 was originally superior to 1, but was not maintained when manual removal of duplicated cases was resulted in non-significant signal**
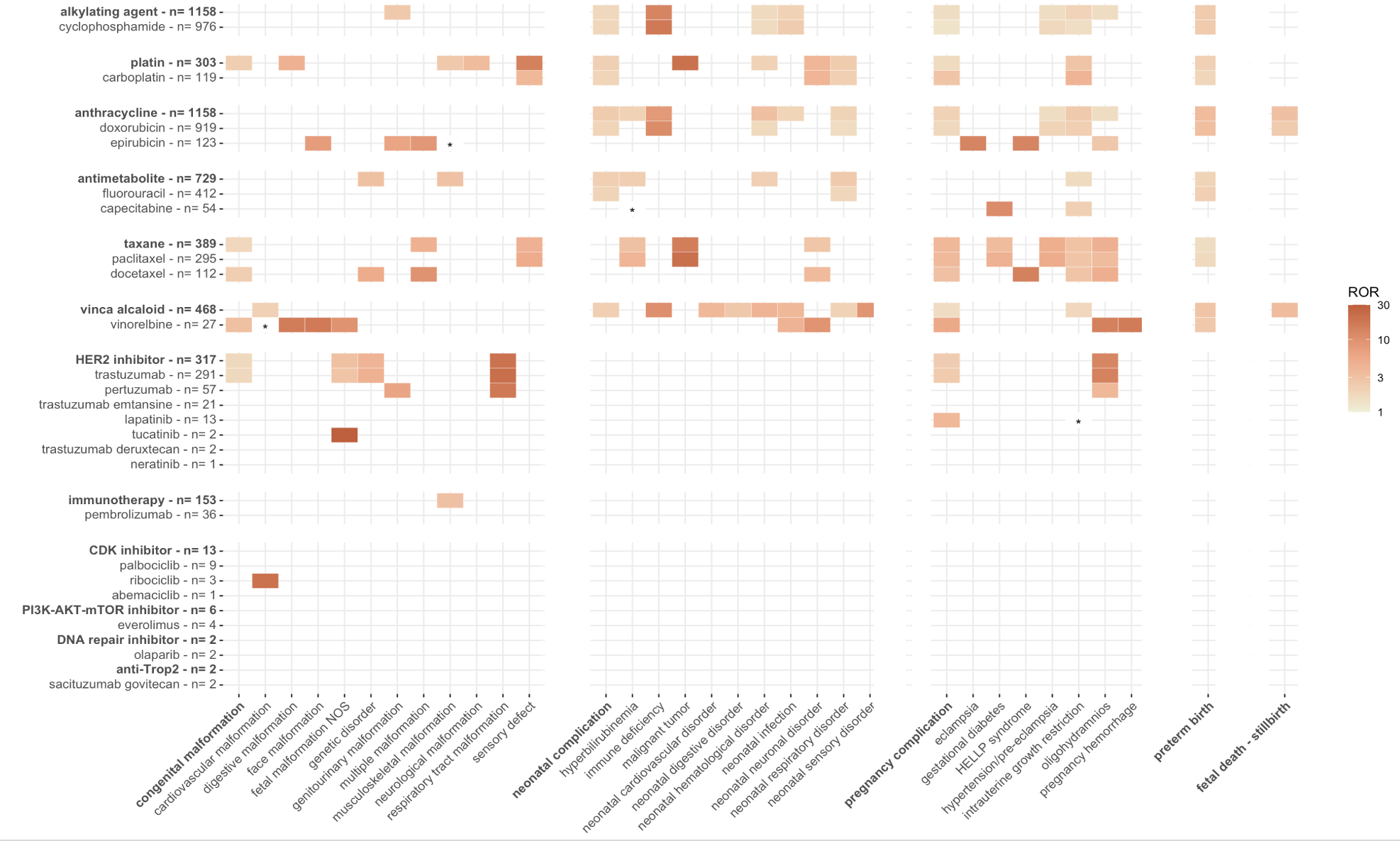
**

## Figure S4. Sensitivity analysis in Breast Cancer only.

Heatmap representing the reporting odds ratio (ROR) of molecules used in breast cancer, restrained to women diagnosed with breast cancer with ROR025 > 1 and with at least two reported cases of any fetal toxicity type.

*ROR025 was originally positive, but was not maintained when manual removal of duplicated cases was resulted in non-significant signal.


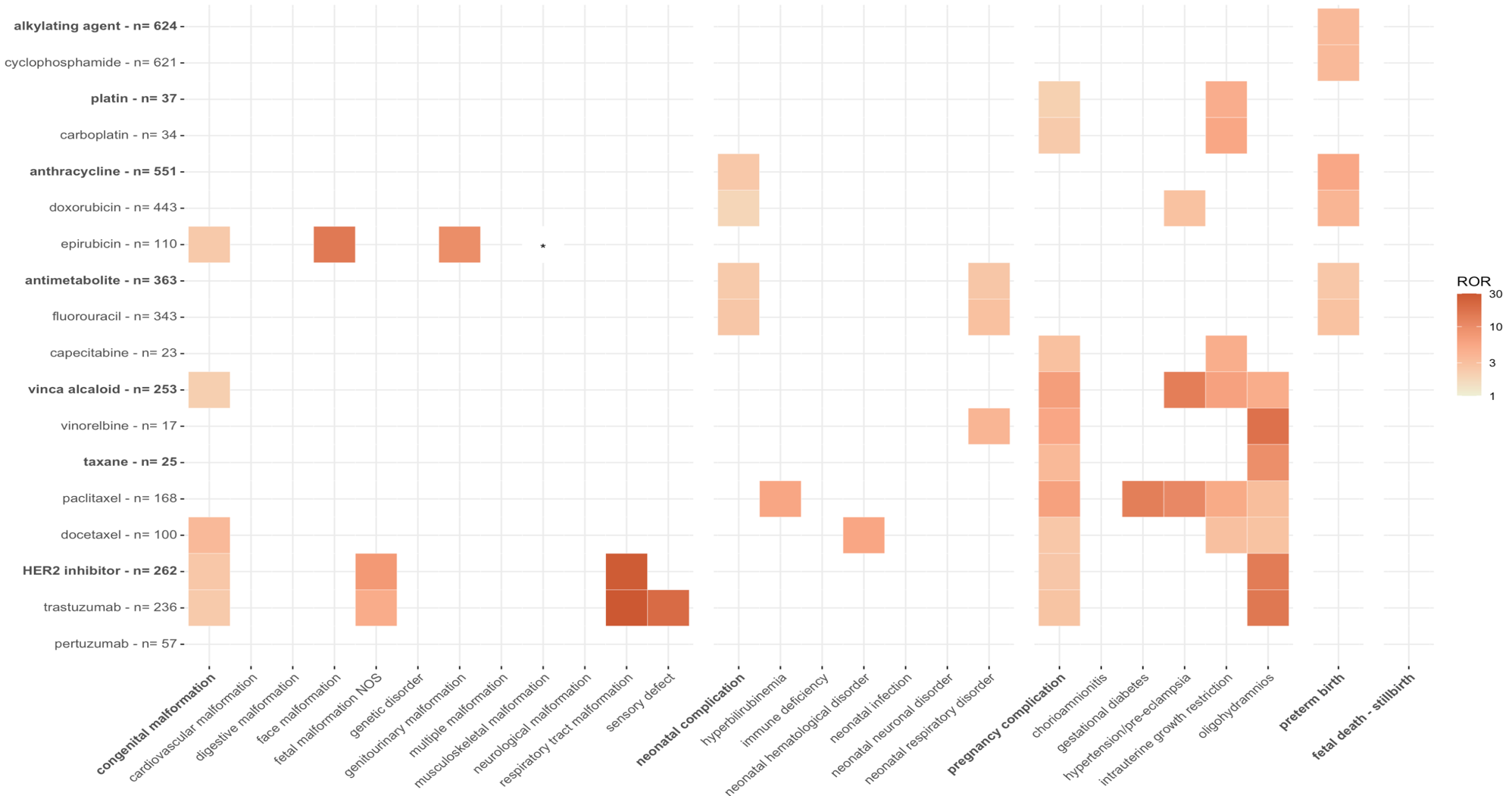


## Figure S5. Sensitivity analysis with cytotoxic-only comparator group.

Heatmap of signals of disproportionate reporting (ROR025 > 1, Nobs ≥ 3) for the four main BC treatments and cyclophosphamide and carboplatin, using a comparator restricted to cytotoxic-only agents (n=262; excluding TKIs and other targeted therapies). Key drug-specific signals observed in the primary analysis were directionally consistent in this sensitivity analysis. ROR, reporting odds ratio; IUGR, intrauterine growth restriction.


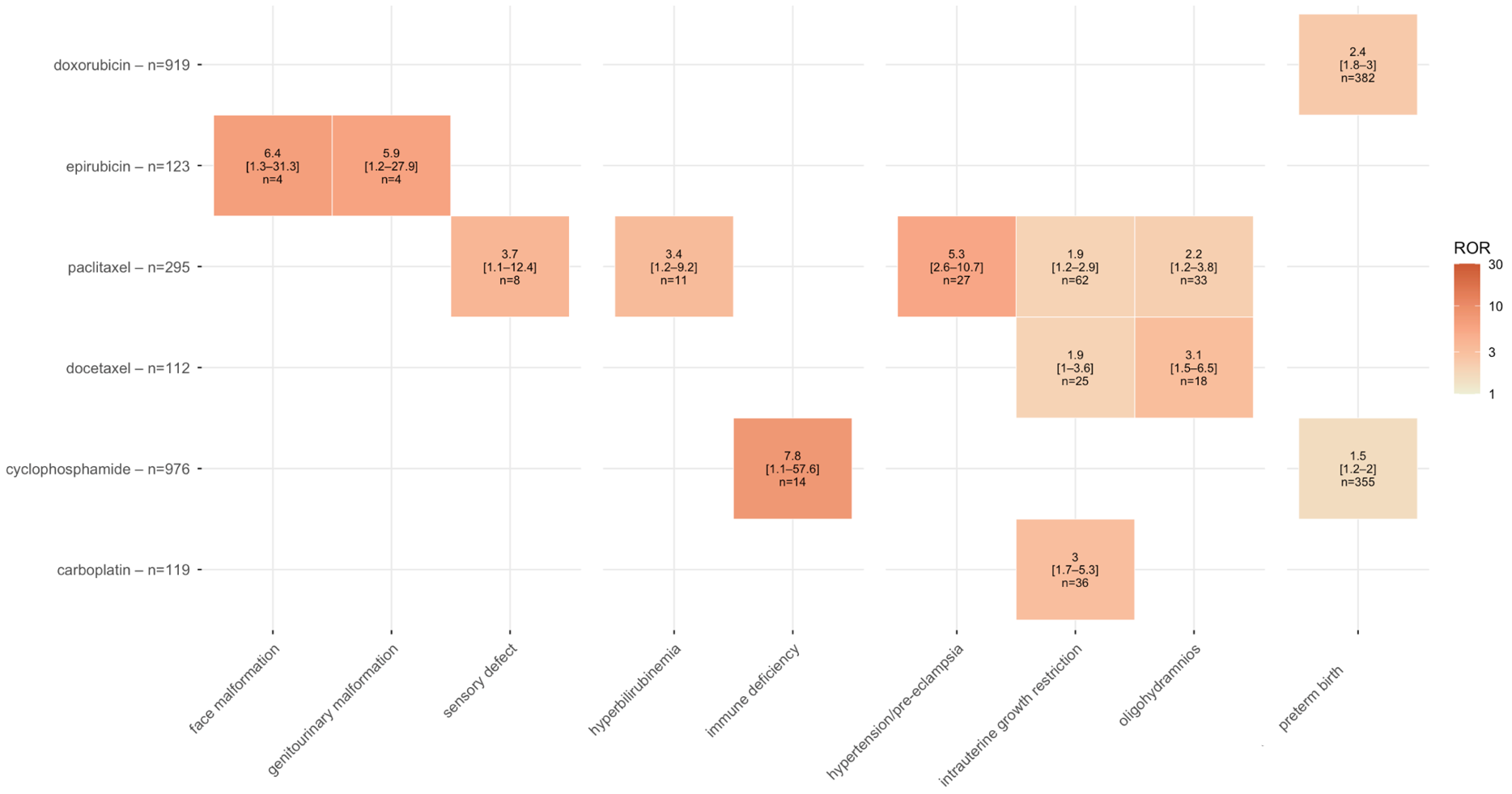


## Figure S6. Anthracycline monotherapy sensitivity analysis.

Forest plot comparing doxorubicin- and epirubicin-associated signals of disproportionate reporting in the primary analysis (which includes cyclophosphamide co-administration) versus a monotherapy-restricted subgroup (reports with anthracycline without cyclophosphamide; doxorubicin n=252, epirubicin n=29). Orange points: ROR025 > 1 and Nobs ≥ 3; grey points: no significant signal. Circles = primary analysis; triangles = cyclophosphamide-free subgroup. The neonatal hematological disorder signal (grey in panel A) was absent in the monotherapy subgroup, supporting a cyclophosphamide-driven mechanism. Wide confidence intervals in the monotherapy subgroup reflect small sample sizes; results are exploratory. ROR, reporting odds ratio; IUGR, intrauterine growth restriction.


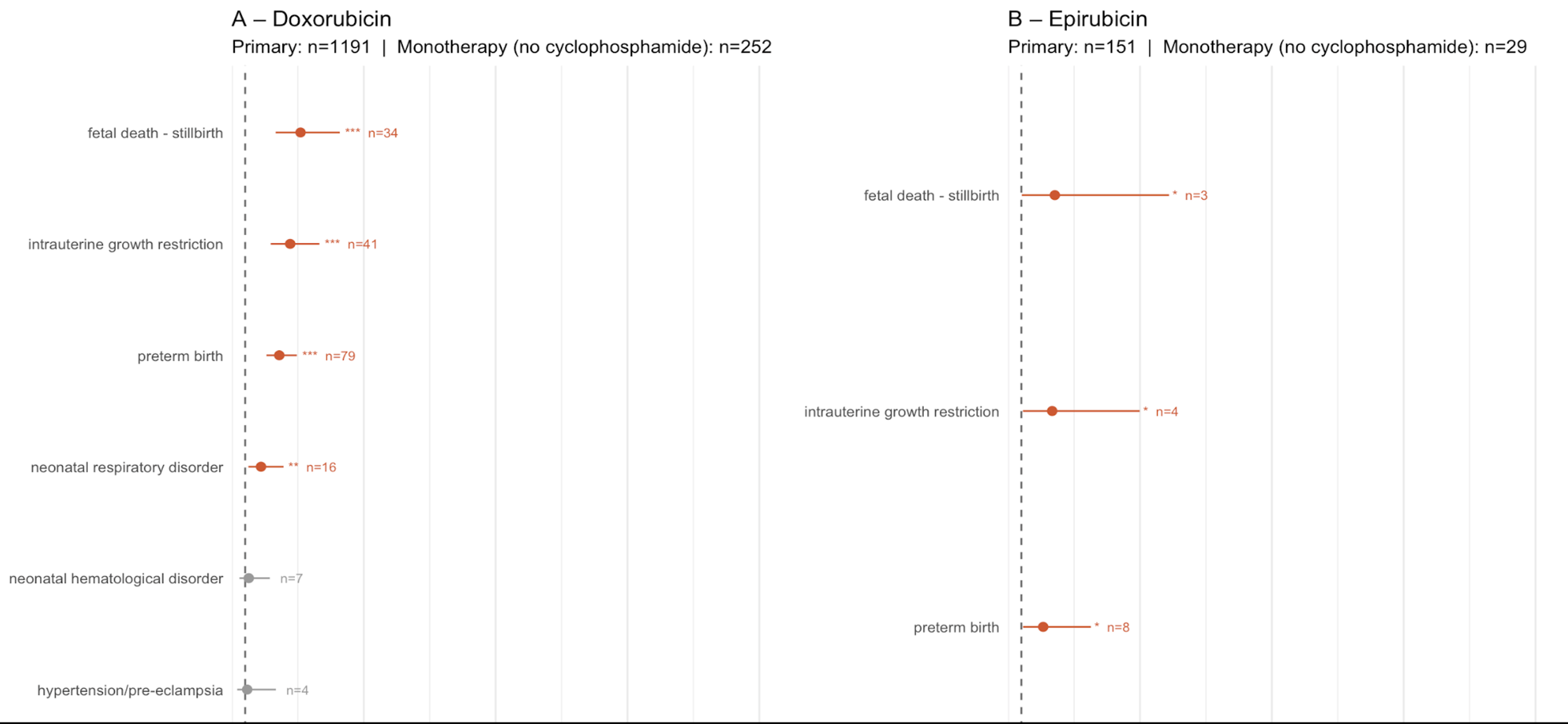


# Supplementary Tables

## Table S1. Detail of VigiBase query.

MedDRA terms used for the initial identification of reports.

| VigiBase data set date | 01/01/2024 |  |
| --- | --- | --- |
| MedDRA version | MedDRA 26.1 (English) |  |
| Search criteria for VigiBase extraction | drugs (ATC group) | L01 ANTINEOPLASTIC AGENTS |
|  | reaction and MedDRA terms | Pregnancy, puerperium and perinatal conditions (SOC) |
|  |  | Fetal and neonatal investigations (HLGT) |
|  |  | Neonatal and perinatal conditions (HLGT) |
|  |  | Neonatal respiratory disorders (HLGT) |
|  |  | Exposures associated with pregnancy, delivery and lactation (HLT) |
|  |  | Fetal therapeutic procedures (HLT) |
|  |  | Induced abortions (HLT) |
|  |  | Obstetric therapeutic procedures (HLT) |
| Number of reports from VigiBase extraction | 10,832 deduplicated cases match your search |  |

## Table S2. Terms corrected.

Terms corrected from initial identification of reports

| MedDRA preferred terms not always associated with pregnancy | |
| --- | --- |
| MedDRA preferred term | mapped as |
| Pelvic girdle pain | Pregnancy symptom |
| Morning sickness | Pregnancy symptom |
| Ghost pregnancy (pseudo embarazo) | Pregnancy symptom |
| Bronchopulmonary dysplasia | Neonatal hypoxic conditions |
| Brief resolved unexplained event | Neonatal hypoxic conditions |
|  |  |
| MedDRA mapping problem |  |
| reported term | mapped as |
| Utero, contracciones | Uterine contractions during pregnancy* |
|  |  |
| Term wrongly encoded |  |
| reported term | mapped as |
| 10049058 | HELLP syndrome** |
| Cervical dilatation | translation problem, cervical dilatation for a neck edema |
|  |  |
| Terms wrongly associated with anticancer drugs | |
| term | mapped as |
| MK-8962 MK-8328 Mk-8415 Mk-9384 | Pembrolizumab |

| *cases of uterus contractions without pregnancy |
| --- |
| **Italian reports link this term to HELLP syndrome, although none are linked to a pregnancy |

## Table S3. MedDRA Preferred Terms used to qualify reports’ exposure type.

MedDRA preferred terms used in reports for the identification of the timing and modality of exposure.

| Exposure type | Preferred Terms |
| --- | --- |
| exposure during pregnancy | Exposure during pregnancy |
|  | First trimester pregnancy |
|  | Foetal exposure during delivery |
|  | Foetal exposure during pregnancy |
|  | High risk pregnancy |
|  | Maternal exposure during delivery |
|  | Maternal exposure during pregnancy |
|  | Pregnancy |
|  | Pregnancy on contraceptive |
|  | Pregnancy on oral contraceptive |
|  | Pregnancy with advanced maternal age |
|  | Pregnancy with contraceptive device |
|  | Pregnancy with injectable contraceptive |
|  | Unintended pregnancy |
|  | Unwanted pregnancy |
| exposure before pregnancy | Drug exposure before pregnancy |
|  | Maternal exposure before pregnancy |
| exposure via breast milk | Exposure via breast milk |
|  | Maternal exposure during breast feeding |
| exposure via semen | Exposure via body fluid |
|  | Exposure via father |
|  | Exposure via partner |
|  | Maternal exposure via partner during pregnancy |
|  | Paternal drugs affecting foetus |
|  | Paternal exposure before pregnancy |
|  | Paternal exposure during pregnancy |
|  | Paternal exposure timing unspecified |
|  | Pregnancy of partner |
| exposure via skin | Accidental exposure to product |
|  | Exposure via direct contact |
|  | Exposure via skin contact |
|  | Occupational exposure to product |

## Table S4. 37 individual materno-fetal adverse outcomes explored

| **outcome category** | **outcome type** |
| --- | --- |
| **congenital malformation** | cardiovascular malformation |
|  | musculoskeletal malformation |
|  | neurological malformation |
|  | fetal malformation not otherwise specified (NOS) |
|  | sensory defect |
|  | digestive malformation |
|  | face malformation |
|  | genitourinary malformation |
|  | genetic disorder |
|  | congenital respiratory tract malformation |
|  | multiple malformation or malformation syndrome |
| **pregnancy complication** | intrauterine growth restriction (IUGR) |
|  | oligohydramnios |
|  | gestational hypertension and pre-eclampsia |
|  | chorioamnionitis |
|  | gestational diabetes |
|  | polyhydramnios |
|  | pregnancy hemorrhage |
|  | hydrops fetalis |
|  | threatened preterm labor |
|  | eclampsia |
|  | HELLP syndrome |
| **preterm birth** | preterm birth |
| **neonatal complication** | neonatal respiratory disorder |
|  | neonatal hematological disorder |
|  | neonatal infection |
|  | hyperbilirubinemia |
|  | neonatal neuronal disorder |
|  | neonatal metabolic - endocrine disorder |
|  | neonatal immune disorder |
|  | neonatal digestive disorder |
|  | neonatal cardiovascular disorder |
|  | neonatal coagulation disorder |
|  | benign tumor |
|  | malignant tumor |
|  | neonatal sensory disorder |
| **fetal death - stillbirth** | fetal death - stillbirth |

## Table S5. Terms deemed not clinically significant.

MedDRA preferred terms deemed not clinically significant when reported alone

| **preferred term reported deemed not clinically significant when reported alone** | **number of occurrences in the whole cohort**  **(n= 3,310)** |
| --- | --- |
| Foetal heart rate abnormal | 12 |
| Foetal hypokinesia | 11 |
| Uterine contractions during pregnancy | 8 |
| Poor feeding infant | 5 |
| Weight decrease neonatal | 5 |
| Foetal heart rate disorder | 3 |
| Large for dates baby | 3 |
| Bradycardia neonatal | 2 |
| Foetal heart rate deceleration abnormality | 2 |
| Foetal heart rate increased | 2 |
| Postmature baby | 2 |
| Uterine contractions abnormal | 2 |
| Agitation neonatal | 1 |
| Foetal arrhythmia | 1 |
| Foetal heart rate decreased | 1 |
| Foetal monitoring abnormal | 1 |
| Phimosis | 1 |
| Poor weight gain neonatal | 1 |
| Tachycardia foetal | 1 |

## Table S6. The Reporting of a Disproportionality Analysis for Drug Safety Signal Detection Using Individual Case Safety Reports in PharmacoVigilance (READUS‑PV) checklist

|  | Item No | Recommendation | Presence in manuscrit | Explication |
| --- | --- | --- | --- | --- |
| Title | 1a | If disproportionality analyses are a prominent component of the published study, the study should be identified as a “disproportionality analysis.” The type of data and name of the database(s) should be specified. | yes | The title clearly identifies the study as a “VigiBase disproportionality analysis study,” specifying both the method (“disproportionality analysis”) and the database (VigiBase), meeting transparency requirements. |
|  | 1b | Report the name of adverse event(s) and/or drug(s) under study, when applicable. | yes | The title states “Maternal and neonatal complications associated with breast cancer systemic treatments,” clearly indicating the drugs (BC systemic treatments) and adverse events (maternal/neonatal complications) under study. |
| Introduction |  |  |  |  |
| Background | 2a | Describe the drug(s) and its utilization, the nature of the adverse event(s) under study and its frequency,  and the existing knowledge on the drug–event combination. | yes | The introduction clearly describes the drugs (breast cancer systemic treatments), their use in pregnancy, and known gaps in safety knowledge. It contextualizes the study by presenting background evidence on pregnancy-associated breast cancer incidence and risks. |
|  | 2b | Specify the rationale for performing the analysis, e.g., as part of routine pharmacovigilance, to investigate an overall safety profile, or to assess a prespecified hypothesis. | yes | The rationale is clearly explained: current safety data are limited, molecule-specific fetal effects remain poorly characterized, and a large-scale pharmacovigilance study is needed to address this knowledge gap. |
|  | 2c | Explain why individual case safety report databases and disproportionality analysis are suitable to fill the  knowledge gap. | yes | The introduction explains that VigiBase contains over 30 million ICSRs, enabling detection of rare but important adverse events. It cites previous pharmacovigilance work as proof of concept for this approach. |
| Objectives | 3 | State specific objectives, identifying the adverse event(s), the drug(s), and the reference group, including any pre-specified hypothesis, if applicable. | yes | The objectives are clearly stated: to evaluate maternal and neonatal outcomes associated with BC systemic treatments, with specific focus on molecule-specific comparisons and trimester-specific risk assessment, using other anticancer treatments as the reference group. |
| Methods |  |  |  |  |
| Study design | 4a | Identify the study (i.e., “disproportionality analysis”) and the type of data used (e.g., “individual case safety reports”). | yes | The methods clearly identify the study as a case/non-case disproportionality analysis using individual case safety reports from VigiBase, the WHO’s global safety database. |
|  | 4b | Provide an outline of the entire study design, including primary and sensitivity analyses performed, and other designs such as case-by-case analysis or literature review. | yes | The methods section outlines the cohort design, primary disproportionality analysis comparing BC treatments with other anticancer treatments, and sensitivity analyses including subgroups (treatment-specific and trimester-specific analyses). It also describes additional analyses such as merging dyads and excluding irrelevant reports. |
| Data description, access,  and pre-processing | 5a | Specify the name of the database(s), the database(s) custodian, and the coverage. Specify the type/number of drugs included within the database and the thesaurus,taxonomies, or ontologies used for coding drugs and events. | yes | VigiBase is specified as the data source, maintained by the Uppsala Monitoring Centre, containing over 30 million ICSRs. Drugs are coded using the Anatomical Therapeutic Classification (ATC) system (group L01), and adverse events using MedDRA v26.1. Coverage of pregnancy-related reactions is explicitly described with detailed MedDRA categories. |
|  | 5b | Specify the extraction dates and describe and justify all choices used for data pre-processing, including any  data transformation or exclusion, if appropriate. | yes | Data were extracted on January 1, 2024. Pre-processing steps are described, including: exclusion of reports unrelated to cancer, removal of duplicates, merging dyads, exclusion based on exposure type, and removing reports with mapping issues. These steps are justified to ensure accurate identification of pregnancy-related adverse events associated with anticancer treatments. |
| Variables definition | 6a | Describe the study population, including any restriction. | yes | The study population consists of ICSRs from VigiBase containing pregnancy-related events and suspect anticancer treatments. Restrictions include exclusion of reports without cancer diagnosis or with non-cancer indications, removal of duplicates, and exclusion of irrelevant exposure types. |
|  | 6b | Describe the nature and the meaning of key variables assessed in the work. | yes | Key variables include: drug exposure (specific BC treatments vs other anticancer drugs), type of adverse event (maternal or fetal/newborn), and timing of exposure during pregnancy. Adverse events are grouped into clinically relevant categories based on MedDRA preferred terms. |
|  | 6c | Specify and justify any grouping of drugs or events. For drugs, specify and justify whether active ingredients/trade names/salts were considered and/or the selected role. |  | BC treatments were grouped based on active ingredients known to be used in pregnancy-associated breast cancer. The “BC treatment” group includes specific active ingredients listed in the methods, while the reference group includes other anticancer treatments. Adverse events were grouped into five clinically relevant categories, with detailed justification provided in supplementary materials. |
|  | 6d | Describe any additional data source used, the type of data, and how they interact with individual case safety reports. | not applicable | No additional data sources were used beyond VigiBase. |
| Statistical methods | 7a | Present any descriptive analysis performed, specifying variables investigated, statistical tests, and significance thresholds. | yes | Descriptive statistics include frequencies for qualitative variables and medians with IQR for quantitative variables. Fisher’s exact test was used for categorical comparisons. Significance threshold was p < 0.05. |
|  | 7b | Describe the measure(s) selected for the disproportionality analysis including any threshold used to identify  signals of disproportionate reporting. Explain the reason for this choice if applicable. | yes | Disproportionality was measured using reporting odds ratio (ROR) with confidence intervals adjusted for multiple testing. Signals were defined as ROR lower bound (ROR025) > 1 with ≥3 occurrences. This is consistent with standard pharmacovigilance signal detection methodology. |
|  | 7c | Clearly describe any sensitivity analysis and any tool to control confounding, including any restriction,  subgroup, stratification, adjustment, or interaction. | yes | Sensitivity analyses included restricting to reports with BC diagnosis, individual analysis for four cornerstone treatments (paclitaxel, docetaxel, doxorubicin, epirubicin), and trimester-specific subgroup analyses. These were performed to assess robustness and confounding. |
|  | 7d | Specify the variables and methods used for the case-by-case analysis, including any algorithm or criteria  used to assess causality, if performed. | yes | Individual case-by-case analysis was performed for reports involving epirubicin, doxorubicin, paclitaxel, and docetaxel. Variables included drug name, trimester of exposure, type of adverse event, and co-medications. Each case was assessed for temporal relationship between drug exposure and event occurrence. No formal algorithm was applied for causality assessment; rather, cases were reviewed qualitatively to identify patterns, trimester-specific associations, and emerging safety signals, as described in the Discussion section. |
|  | 7e | Specify any statistical methods used for other data sources. | not applicable | No additional data sources were used. |
| Results |  |  |  |  |
| Participants | 8a | Specify the number of individual case safety reports included at each stage, including reasons for exclusion. | yes | **Number of reports at each stage:** - Extracted: 10,832 deduplicated reports  - Retained for analysis: 3,310 reports of pregnant individuals exposed to anticancer treatments    • BC treatment group: 1,789 reports    • Other anticancer treatment group: 1,521 reports  **Exclusions:** Reports not involving pregnant individuals exposed to anticancer treatments, duplicates removed. |
|  | 8b | Provide key demographic and clinical characteristics of cases, if possible comparing cases with any appropriate  reference group. | yes | **Demographics and clinical characteristics:** - Mean age: BC group 27.4 (SD 13.8) vs Other group 29.3 (SD 11.7) years.  - Cancer type (BC group): breast cancer 908 (58%), lymphoma 298 (19%), sarcoma 72 (5%).  - Cancer type (Other group): chronic myeloid leukemia 727 (56%), acute leukemia 156 (12%), lymphoma 54 (4%).  - Main anticancer agents: cyclophosphamide, doxorubicin, fluorouracil, paclitaxel, trastuzumab, epirubicin, docetaxel, carboplatin (BC group); cytarabine, daunorubicin, cisplatin (Other group). |
| Disproportionality analysis | 9 | Present all results including confidence intervals. Present also results of sensitivity analyses, if performed. | yes | **Key results:** - Pregnancy/fetal outcomes: BC group 998 reports (55.8%), Other group 470 reports (30.9%).  - Total fetal toxicities: BC group 1,595; Other group 783; **ROR=2.8 [95%CI=2.4–3.2], p<10^-3**.  - Specific drug-class toxicities:    • Cyclophosphamide: neonatal immunodeficiency (ROR 17.0 [3.8–75]), hematological disorders (ROR ~1.6–1.7).    • Doxorubicin: neonatal immunodeficiency (ROR 11.0 [3.2–40]), IUGR (ROR 2.1 [1.7–2.7]), stillbirth (ROR 2.4 [1.7–3.4]).    • Taxanes: IUGR (ROR ~2.8–2.9), oligohydramnios (ROR ~3.5–4.9), pre-eclampsia (ROR up to 5.2 [3.3–8.4]).    • Carboplatin: neuronal disorders (ROR 4.2 [1.7–10]), sensory defects (ROR 3.9 [1.2–13.3]).    • Trastuzumab: oligohydramnios (ROR 14.0 [9.8–20]).  - Trimester analysis (n=221): first trimester exposures had higher adverse outcomes: facial malformations ROR=32.7 [5.2–205.6], musculoskeletal malformations ROR=20.9 [2.8–154.6], sensory defects ROR=20.0 [1.2–329.8], neonatal neuronal disorders ROR=8.3 [1.5–45.0], oligohydramnios ROR=6.9 [2.1–22.8], preterm birth ROR=2.7 [1.2–6.32], all p<0.05. |
| Case-by-case analysis | 10 | Present the case-by-case analysis of key variables. Present the causality assessment, if applicable. | yes | - Detailed analysis by molecule, drug class, and trimester.  - Strong drug-specific adverse event patterns identified.  - Causality: No causality assessment done.  ROR values provided, showing statistically significant associations between specific drugs and adverse pregnancy/neonatal outcomes (see above).  - Sensitivity analysis (BC subpopulation, n=908) confirmed main findings: cyclophosphamide and doxorubicin linked to preterm birth, epirubicin linked to congenital malformations, taxanes linked to vascular-placental toxicities, trastuzumab linked to oligohydramnios and respiratory malformations. |
| Discussion |  |  |  |  |
| Key results | 11 | Discuss key results with reference to study objectives and contextualize them within the current literature  and other consulted sources. Clearly discriminate between expected reactions and emerging safety  signals. | yes | - Analysis of >3,000 reports of anticancer drug exposure during pregnancy showed significantly higher maternal–fetal adverse outcome reporting in BC treatments (55.8%) vs other anticancer treatments (30.9%).  - Distinct drug-specific and class-specific patterns were observed:   • Anthracyclines (especially doxorubicin) linked to hematological, immune, respiratory disorders, pre‑eclampsia, IUGR, stillbirth; no significant overreporting of cardiovascular malformations.   • Epirubicin associated with facial and genitourinary malformations — a novel signal not previously reported.   • Cyclophosphamide showed hematologic and immune complications, IUGR, preterm birth; frequent co‑administration with anthracyclines complicates attribution.   • Taxanes (especially paclitaxel) showed sensory defects, hyperbilirubinemia, neurologic disorders, gestational diabetes, pre‑eclampsia, IUGR, oligohydramnios.   • Carboplatin associated with sensory, neuronal, respiratory disorders despite limited literature.   • Capecitabine linked to IUGR; anti-HER2 therapy associated with oligohydramnios and respiratory malformations.   • First-trimester exposures showed higher adverse outcomes, particularly facial malformations with epirubicin. |
| External validity | 12a | Discuss the external validity of the results to the general population. | yes | - The findings are broadly applicable to pregnant women receiving BC treatment, especially in tertiary care settings where multidrug regimens are used.  - Data drawn from Vigibase encompass international reports, supporting generalizability, but reporting biases and variability in healthcare practices must be considered. |
|  | 12b | Discuss the potential relevance of results in clinical practice | yes | - First-trimester chemotherapy should be avoided where possible.  - Anthracycline–cyclophosphamide regimens should be timed away from delivery to reduce neonatal neutropenia.  - Taxanes may be safer in peri-delivery periods, though caution is warranted for sensory and neurological complications.  - Findings emphasize drug‑ and trimester-specific risks and support multidisciplinary treatment planning involving oncology, obstetrics, neonatology, and pharmacology teams.  - Close fetal and neonatal monitoring should be tailored to the agent used. |
|  | 12c | Propose further study designs if applicable | not applicable | - Not applicable in this study, though we note need for prospective registries and cohort studies to assess trimester-specific and dose-dependent risks, long-term neurodevelopmental outcomes, and emerging therapies. |
| Limitations | 13 | Present general limitations, making clear that disproportionality analysis alone cannot prove causation  or measure incidence, and specific limitations, including confounding and reporting bias and efforts to mitigate them. | yes | - Intrinsic reporting bias: adverse events more likely reported if severe/unexpected → potential overestimation of risk.  - Incomplete clinical data: dose, gestational age, pregnancy outcomes often missing.  - Confounding by indication and co‑medication could not be adjusted.  - Causality cannot be confirmed from disproportionality analysis.  - No long-term follow-up data on exposed children.  - Limited literature on some agents (e.g., carboplatin, epirubicin, anti‑HER2 agents, newer targeted therapies). |
| Declarations |  |  |  |  |
|  | 14a | Provide the source of funding/sponsorship and the role of the funders/sponsors for the present study and for any original study on which the present article is based. | yes | Source of funding explicitely written in the manuscrit |
|  | 14b | Clearly identify potential commercial and intellectual conflicts of interest (e.g., link to any drug/event  investigated, whether financial, legal action, or software used). | yes | Statement explicitely written in the manuscrit |
|  | 14c | Declare any institutional approval needed or granted in the investigation. | yes | No additional institutional approval was required for this pharmacovigilance study. Data were extracted from the publicly available Vigibase database. All data use complied with applicable institutional guidelines and regulations. |
|  | 14d | Include a statement on data availability, code availability (including the version of the statistical software  used), and protocol registration. | yes | Publicly available datasets were analyzed in this study. Data can be accessed at: [https://www.vigiaccess.org](https://www.vigiaccess.org/). The study has not been published or presented elsewhere. Statistical analyses were conducted using R software (version details available upon request). The study protocol was not registered. |

­

## Table S7. The Strengthening the Reporting of Observational Studies in Epidemiology (STROBE) checklist: guidelines for reporting observational studies.

|  | Item No | Recommendation | Presence in manuscrit | Explication |
| --- | --- | --- | --- | --- |
| Title and abstract | 1 | (a) Indicate the study's design with a commonly used term in the Title or the abstract | yes | Disproportionality analysis study using VigiBase pharmacovigilance data |
|  |  | (b) Provide in the abstract an informative and balanced summary of what was done and what was found | yes | Analyzed VigiBase reports to assess maternal and neonatal complications of breast cancer systemic treatments during pregnancy, identifying treatment-specific safety signals. |
| Introduction |  |  |  |  |
| Background/rationale | 2 | Explain the scientific background and rationale for the investigation being reported | yes | Breast cancer treatment during pregnancy is rare and under-researched; safety data are limited, requiring pharmacovigilance analysis. |
| Objectives | 3 | State specific objectives, including any prespecified hypotheses | yes | To investigate maternal and neonatal outcomes associated with breast cancer systemic treatments during pregnancy using VigiBase data. |
| Methods |  |  |  |  |
| Study design | 4 | Present key elements of study design early in the paper | yes | Retrospective pharmacovigilance disproportionality analysis of VigiBase reports. |
| Setting | 5 | Describe the setting, locations, and relevant dates, including periods of recruitment, exposure, follow-up, and data collection | yes | Data extracted from VigiBase (WHO global database of individual case safety reports), up to 2024. |
| Participants | 6 | (a) Give the eligibility criteria, and the sources and methods of case ascertainment and control selection. Give the rationale for the choice of cases and controls | yes | Included all reports of breast cancer systemic treatments in pregnancy with maternal or neonatal outcomes in VigiBase. |
|  |  | (b) For matched studies, give matching criteria and the number of controls per case | not applicable |  |
| Variables | 7 | Clearly define all outcomes, exposures, predictors, potential confounders, and effect modifiers. Give diagnostic criteria, if applicable | yes | Outcomes: maternal and neonatal complications; exposures: specific chemotherapeutics; confounders: trimester, co-medications. |
| Data sources/ measurement | 8* | For each variable of interest, give sources of data and details of methods of assessment (measurement). Describe comparability of assessment methods if there is more than one group | yes | Data from VigiBase; adverse events coded using MedDRA; exposure details extracted from reports. |
| Bias | 9 | Describe any efforts to address potential sources of bias | yes | Possible underreporting and reporting bias addressed by sensitivity analysis and case-by-case review. |
| Study size | 10 | Explain how the study size was arrived at | yes | Included all eligible cases from VigiBase at time of query. |
| Quantitative variables | 11 | Explain how quantitative variables were handled in the analyses. If applicable, describe which groupings were chosen and why | yes | Numbers of reports and reporting odds ratios computed; trimester and drug grouping applied. |
| Statistical methods | 12 | (a) Describe all statistical methods, including those used to control for confounding | yes | Disproportionality analysis with reporting odds ratio; subgroup analysis by drug and trimester. |
|  |  | (b) Describe any methods used to examine subgroups and interactions | yes | Subgroup analysis by drug class and trimester. |
|  |  | (c) Explain how missing data were addressed | yes | Cases with incomplete exposure/outcome details included in qualitative analysis. |
|  |  | (d) If applicable, explain how matching of cases and controls was addressed | not applicable |  |
|  |  | (e) Describe any sensitivity analyses | yes | Conducted to assess robustness of disproportionality results and potential confounders. |
| Results |  |  |  |  |
| Participants | 13* | (a) Report numbers of individuals at each stage of study—eg numbers potentially eligible, examined for eligibility, confirmed eligible, included in the study, completing follow-up, and analysed | yes | All eligible cases of breast cancer systemic treatment in pregnancy extracted from VigiBase were included; numbers detailed in Results and flow diagram. |
|  |  | (b) Give reasons for non-participation at each stage | yes | Excluded cases lacked pregnancy exposure or outcome data, or were duplicates. |
|  |  | (c) Consider use of a flow diagram | yes | A flow diagram summarising case selection is included. |
| Descriptive data | 14* | (a) Give characteristics of study participants (eg demographic, clinical, social) and information on exposures and potential confounders | yes | Demographics, treatment regimens, pregnancy trimester, maternal and neonatal outcomes presented in Table 1 and supplementary material. |
|  |  | (b) Indicate number of participants with missing data for each variable of interest | yes | Missing data for key variables reported in Table S3. |
| Outcome data | 15* | Report numbers in each exposure category, or summary measures of exposure | yes | Number of reports per drug class and trimester presented in results and figures. |
| Main results | 16 | (a) Give unadjusted estimates and, if applicable, confounder-adjusted estimates and their precision (eg, 95% confidence interval). Make clear which confounders were adjusted for and why they were included | yes | Reporting Odds Ratios with 95% CI for drug-outcome associations; adjusted for potential confounders where applicable. |
|  |  | (b) Report category boundaries when continuous variables were categorized | yes | Trimester grouped as first, second, third; drug classes defined by ATC codes. |
|  |  | (c) If relevant, consider translating estimates of relative risk into absolute risk for a meaningful time period | not applicable |  |
| Other analyses | 17 | Report other analyses done—eg analyses of subgroups and interactions, and sensitivity analyses | yes | Subgroup analyses by trimester, drug class; sensitivity analyses excluding incomplete cases performed. |
| Discussion |  |  |  |  |
| Key results | 18 | Summarise key results with reference to study objectives | yes | Identified maternal and neonatal safety signals related to systemic breast cancer treatments in pregnancy, varying by drug class and trimester. |
| Limitations | 19 | Discuss limitations of the study, taking into account sources of potential bias or imprecision. Discuss both direction and magnitude of any potential bias | yes | Limitations include underreporting, reporting bias, missing data, and lack of causality inference; direction and magnitude of bias discussed. |
| Interpretation | 20 | Give a cautious overall interpretation of results considering objectives, limitations, multiplicity of analyses, results from similar studies, and other relevant evidence | yes | Findings highlight potential safety signals requiring further investigation in dedicated prospective studies. |
| Generalisability | 21 | Discuss the generalisability (external validity) of the study results | yes | Results apply to pharmacovigilance database context and may not reflect absolute risk; external validity limited by reporting bias. |
| Other information |  |  |  |  |
| Funding | 22 | Give the source of funding and the role of the funders for the present study and, if applicable, for the original study on which the present article is based | yes | Funded by “Contrats ED: Programme blanc Institut Curie PSL” and “SHS INCa”; funders had no role in study design, data collection, analysis, interpretation, or writing. |

## Table S8. Number of reports and type of anticancer involved in the BC treatments exposure group (n = 1,789), with number of molecules received by patient

| **Molecule reported** | **Overall - N(%)** |
| --- | --- |
| cyclophosphamide | 976 (54.6) |
| paclitaxel | 295 (16.5) |
| docetaxel | 112 (6.3) |
| epirubicin | 123 (6.9) |
| doxorubicin | 919 (51.4) |
| carboplatin | 119 (6.7) |
| capecitabine | 54 (3.0) |
| vinorelbine | 27 (1.5) |
| fluorouracil | 412 (23.0) |
| everolimus | 4 (0.2) |
| sacituzumab govitecan | 2 (0.1) |
| pembrolizumab | 36 (2.0) |
| trastuzumab | 291 (16.3) |
| tucatinib | 2 (0.1) |
| neratinib | 1 (0.1) |
| trastuzumab emtansine | 21 (1.2) |
| trastuzumab deruxtecan | 2 (0.1) |
| lapatinib | 13 (0.7) |
| pertuzumab | 57 (3.2) |
| palbociclib | 9 (0.5) |
| abemaciclib | 1 (0.1) |
| ribociclib | 3 (0.2) |
| olaparib | 2 (0.1) |
| **Number of anticancer treatments received by patient** |  |
| 1 | 763 (69.3) |
| 2 | 512 (46.5) |
| 3 | 416 (37.8) |
| 4 or more | 98 (8.9) |

## Table S9. Number of reports and type of molecule involved in the other anticancer treatment group n = 1,521)

*We restricted the number of molecules reported 10 or more times for readability. Molecules could be administered in monotherapy or in combination with another one.*

*Percentages represent the proportion of each molecule divided by the total number of patients in the other anticancer treatment group (n=1521).*

| **Molecule** | **Number of reports**  **N(%)** |
| --- | --- |
| imatinib | 630 (41.4) |
| nilotinib | 215 (14.1) |
| dasatinib | 127 (8.3) |
| cytarabine | 78 (5.1) |
| daunorubicin | 59 (3.9) |
| cisplatin | 55 (3.6) |
| tretinoin | 51 (3.4) |
| nivolumab | 48 (3.2) |
| hydroxycarbamide | 42 (2.8) |
| idarubicin | 42 (2.8) |
| etoposide | 35 (2.3) |
| vincristine | 33 (2.2) |
| methotrexate | 32 (2.1) |
| rituximab | 32 (2.1) |
| lenalidomide | 30 (2) |
| bleomycin | 24 (1.6) |
| anagrelide | 23 (1.5) |
| temozolomide | 22 (1.4) |
| ipilimumab | 20 (1.3) |
| interferon | 19 (1.2) |
| alectinib | 16 (1.1) |
| mercaptopurine | 14 (0.9) |
| fludarabine | 12 (0.8) |
| interferon alfa | 12 (0.8) |
| brentuximab vedotin | 11 (0.7) |
| erlotinib | 11 (0.7) |
| bortezomib | 10 (0.7) |
| mitoxantrone | 10 (0.7) |

## Table S10. Description of all pregnancy and fetal/newborn adverse outcomes among patients who received molecules used in BC treatments exposure group and other anticancer treatment group

| **Fetal toxicity type** | **BC treatments exposure group (total reports (n=1789)  Number of occurrence**  **N (%)** | **Other anticancer treatment group total reports (n=1521)  Number of occurrence**  **N (%)** |
| --- | --- | --- |
| **Number of different reports** | **998** | **470** |
| **Total number of fetal toxicity** | **1595** | **783** |
| preterm birth | 566 (35.5) | 241 (30.8) |
| intrauterine growth restriction (IUGR) | 241 (15.1) | 86 (11) |
| oligohydramnios | 124 (7.8) | 15 (1.9) |
| neonatal respiratory disorder | 111 (7) | 59 (7.5) |
| fetal death - stillbirth | 80 (5) | 59 (7.5) |
| neonatal hematological disorder | 60 (3.8) | 43 (5.5) |
| gestational HT and pre-eclampsia | 57 (3.6) | 27 (3.4) |
| cardiovascular malformation | 46 (2.9) | 35 (4.5) |
| neonatal infection | 41 (2.6) | 19 (2.4) |
| neonatal neuronal disorder | 30 (1.9) | 16 (2.0) |
| hyperbilirubinemia | 27 (1.7) | 12 (1.5) |
| musculoskeletal malformation | 25 (1.6) | 15 (1.9) |
| fetal malformation NOS | 17 (1.1) | 12 (1.5) |
| neonatal metabolic - endocrine disorder | 17 (1.1) | 13 (1.7) |
| neonatal immune disorder | 16 (1) | 0 (0.0) |
| neonatal cardiovascular disorder | 15 (0.9) | 3 (0.4) |
| sensory defect | 13 (0.8) | 11 (1.4) |
| neurological malformation | 12 (0.8) | 11 (1.4) |
| neonatal digestive disorder | 11 (0.7) | 9 (1.1) |
| face malformation | 10 (0.6) | 8 (1) |
| genitourinary malformation | 10 (0.6) | 11 (1.4) |
| digestive malformation | 9 (0.6) | 10 (1.3) |
| genetic disorder | 7 (0.4) | 6 (0.8) |
| gestational diabetes | 7 (0.4) | 6 (0.8) |
| congenital respiratory tract malformation | 6 (0.4) | 3 (0.4) |
| neonatal effusion | 5 (0.3) | 7 (0.9) |
| pregnancy hemorrhage | 5 (0.3) | 4 (0.5) |
| multiple malformation or malformation syndrome | 4 (0.3) | 4 (0.5) |
| benign tumor | 4 (0.3) | 5 (0.6) |
| neonatal coagulation disorder | 3 (0.2) | 7 (0.9) |
| malignant tumor | 3 (0.2) | 0 (0) |
| polyhydramnios | 3 (0.2) | 6 (0.8) |
| neonatal sensory disorder | 2 (0.1) | 3 (0.4) |
| HELLP syndrome | 2 (0.1) | 1 (0.1) |
| chorioamnionitis | 2 (0.1) | 6 (0.8) |
| threatened preterm labor | 2 (0.1) | 3 (0.4) |
| hydrops fetalis | 1 (0.1) | 5 (0.6) |
| eclampsia | 1 (0.1) | 2 (0.3) |

## Table S11: Reporting Odds Ratios (ROR) of Fetal Toxicity by Drug and Adverse Drug Reaction (ADR) for all molecules or classes and all ADR

Nobs: Number of observed cases

Cell Values: If Nobs > 0: ROR [ROR025-ROR975] ; Nobs = x

ROR: Reporting Odds Ratio for the ADR with the drug.

If Nobs = 0: "0" indicates no cases observed.

The following seven molecules/classes (trastuzumab deruxtecan, neratinib, abemaciclib, DNA repair inhibitor, olaparib, anti-Trop2, sacituzumab govitecan) were excluded from this table because no cases of any ADR were observed.

| **ADR** | **alkylating agent**  **N=1158** | **Cyclophosphamide**  **N=976** | **Platin**  **N=303** | **Carboplatin**  **N=119** | **Anthracycline**  **N=1158** | **Doxorubicin**  **N=919** | **Epirubicin**  **N=123** | **Antimetabolite**  **N=729** | **Fluorouracil**  **N=412** | **Capecitabine**  **N=54** | **Taxane**  **N=389** | **Paclitaxel**  **N=295** | **Docetaxel**  **N=112** |
| --- | --- | --- | --- | --- | --- | --- | --- | --- | --- | --- | --- | --- | --- |
| **congenital malformation**  **N=223** | 0.8[0.6-1.1] n=70 | 0.8[0.6-1.1] n=55 | 2.3[1.6-3.3] n=39 | 1.7[1.0-3.1] n=13 | 1.0[0.7-1.3] n=77 | 0.8[0.5-1.0] n=51 | 1.7[0.9-3.0] n=13 | 0.9[0.6-1.2] n=44 | 0.5[0.3-0.9] n=16 | 0.8[0.3-2.6] n=3 | 1.8[1.2-2.5] n=41 | 1.4[0.9-2.1] n=26 | 2.2[1.3-3.9] n=15 |
| **cardiovascular malformation**  **N=81** | 0.9[0.6-1.5] n=27 | 0.8[0.5-1.4] n=21 | 1.1[0.5-2.3] n=8 | 1.0[0.3-3.3] n=3 | 1.4[0.9-2.2] n=35 | 1.0[0.6-1.6] n=22 | 1.4[0.5-3.8] n=4 | 1.0[0.6-1.7] n=18 | 0.5[0.2-1.1] n=5 | 0.7[0.1-5.5] n=1 | 0.8[0.4-1.7] n=8 | 0.5[0.2-1.4] n=4 | 1.5[0.5-4.2] n=4 |
| **digestive malformation**  **N=19** | 0.5[0.2-1.5] n=4 | 0.6[0.2-1.9] n=4 | 4.7[1.8-12.3] n=6 | 1.5[0.2-11.3] n=1 | 0.5[0.2-1.5] n=4 | 0.5[0.1-1.7] n=3 | 1.4[0.2-10.9] n=1 | 0.9[0.3-2.9] n=4 | 0 | 0 | 0 | 0 | 0 |
| **face malformation**  **N=18** | 1.9[0.7-4.7] n=9 | 1.5[0.6-3.9] n=7 | 1.2[0.3-5.4] n=2 | 0 | 1.5[0.6-3.8] n=8 | 0.5[0.1-1.8] n=3 | 7.6[2.5-23.5] n=4 | 1.8[0.7-4.8] n=6 | 1.4[0.4-4.9] n=3 | 0 | 0.4[0.1-3.3] n=1 | 0.6[0.1-4.5] n=1 | 0 |
| **fetal malformation NOS**  **N=29** | 0.5[0.2-1.2] n=6 | 0.3[0.1-0.9] n=3 | 2.1[0.8-5.5] n=5 | 1.0[0.1-7.1] n=1 | 0.4[0.1-1.0] n=5 | 0.5[0.2-1.4] n=5 | 0 | 1.1[0.5-2.7] n=7 | 1.1[0.4-3.3] n=4 | 2.2[0.3-16.3] n=1 | 1.6[0.6-4.1] n=5 | 1.2[0.4-3.9] n=3 | 2.1[0.5-9.1] n=2 |
| **genetic disorder**  **N=13** | 0.8[0.3-2.7] n=4 | 0.4[0.1-2.0] n=2 | 3.0[0.8-10.9] n=3 | 2.2[0.3-17.4] n=1 | 1.2[0.4-3.6] n=5 | 0.8[0.2-2.8] n=3 | 0 | 3.1[1.0-9.1] n=6 | 1.3[0.3-5.8] n=2 | 0 | 1.4[0.3-6.2] n=2 | 0 | 5.3[1.2-24.1] n=2 |
| **genitourinary malformation**  **N=21** | 2.5[1.0-5.9] n=12 | 2.2[0.9-5.2] n=10 | 1.7[0.5-5.7] n=3 | 1.3[0.2-10.1] n=1 | 1.7[0.7-4.0] n=10 | 0.8[0.3-2.2] n=5 | 6.3[2.1-18.9] n=4 | 0.6[0.2-2.0] n=3 | 0 | 0 | 1.8[0.6-5.3] n=4 | 1.1[0.2-4.6] n=2 | 3.0[0.7-13.2] n=2 |
| **multiple malformation**  **N=8** | 3.1[0.7-13.0] n=5 | 1.4[0.3-6.0] n=3 | 1.4[0.2-11.6] n=1 | 0 | 1.1[0.3-4.7] n=3 | 0.4[0.0-3.0] n=1 | 8.8[1.8-43.9] n=2 | 2.1[0.5-8.9] n=3 | 2.4[0.5-11.7] n=2 | 0 | 4.5[1.1-19.0] n=3 | 1.5[0.2-11.9] n=1 | 9.7[1.9-48.5] n=2 |
| **musculoskeletal malformation**  **N=40** | 1.4[0.7-2.6] n=17 | 1.0[0.5-2.0] n=12 | 2.5[1.2-5.5] n=8 | 2.2[0.7-7.3] n=3 | 1.5[0.8-2.9] n=18 | 1.1[0.6-2.2] n=12 | 2.9[1.0-8.4] n=4 | 2.9[1.6-5.5] n=18 | 1.8[0.8-3.9] n=8 | 0 | 1.1[0.4-2.8] n=5 | 1.1[0.4-3.2] n=4 | 0.7[0.1-5.4] n=1 |
| **neurological malformation**  **N=23** | 1.2[0.5-2.8] n=9 | 0.8[0.3-2.1] n=6 | 3.6[1.4-9.1] n=6 | 1.2[0.2-9.1] n=1 | 1.7[0.8-3.9] n=11 | 2.0[0.9-4.6] n=10 | 0 | 1.0[0.4-2.7] n=5 | 0.3[0.0-2.4] n=1 | 0 | 0.7[0.2-3.1] n=2 | 0.5[0.1-3.4] n=1 | 1.3[0.2-9.7] n=1 |
| **respiratory tract malformation**  **N=9** | 0.5[0.1-2.6] n=2 | 0.7[0.1-3.3] n=2 | 0 | 0 | 0.2[0.0-1.9] n=1 | 0 | 3.3[0.4-26.2] n=1 | 0 | 0 | 0 | 0.9[0.1-7.5] n=1 | 1.3[0.2-10.3] n=1 | 0 |
| **sensory defect**  **N=24** | 0.4[0.1-1.1] n=4 | 0.3[0.1-1.1] n=3 | 14.5[6.4-33.0] n=14 | 3.9[1.1-13.3] n=3 | 0.4[0.1-1.1] n=4 | 0.2[0.1-1.0] n=2 | 0 | 0.7[0.2-2.1] n=4 | 0 | 2.7[0.4-20.0] n=1 | 4.6[2.0-10.6] n=9 | 5.2[2.2-12.3] n=8 | 1.2[0.2-9.3] n=1 |
| **neonatal complication**  **N=402** | 1.7[1.4-2.1] n=187 | 1.8[1.4-2.2] n=164 | 2.1[1.6-2.8] n=64 | 1.9[1.2-3.0] n=24 | 2.4[1.9-2.9] n=214 | 1.9[1.5-2.3] n=160 | 1.5[0.9-2.5] n=21 | 2.2[1.7-2.7] n=142 | 1.9[1.5-2.5] n=80 | 0.7[0.3-1.9] n=5 | 1.2[0.9-1.7] n=55 | 1.1[0.8-1.6] n=40 | 1.4[0.8-2.3] n=18 |
| **benign tumor**  **N=9** | 0.9[0.2-3.7] n=3 | 0.7[0.1-3.3] n=2 | 2.8[0.6-13.8] n=2 | 3.4[0.4-27.2] n=1 | 1.5[0.4-5.6] n=4 | 0.7[0.2-3.6] n=2 | 3.3[0.4-26.2] n=1 | 1.8[0.4-7.1] n=3 | 0 | 0 | 2.2[0.4-10.4] n=2 | 2.9[0.6-14.2] n=2 | 0 |
| **Hyperbilirubinemia**  **N=39** | 1.0[0.5-2.0] n=14 | 0.8[0.4-1.7] n=10 | 2.2[1.0-5.0] n=7 | 1.5[0.3-6.1] n=2 | 2.0[1.0-3.7] n=20 | 1.8[1.0-3.5] n=16 | 0.7[0.1-5.0] n=1 | 2.2[1.2-4.3] n=15 | 1.5[0.7-3.5] n=7 | 5.3[1.6-17.6] n=3 | 3.0[1.5-6.1] n=11 | 4.1[2.0-8.4] n=11 | 0 |
| **immune deficiency**  **N=16** | 13.2[3.0-58.0] n=14 | 17.0[3.8-74.8] n=14 | 1.4[0.3-6.3] n=2 | 0 | 8.1[2.3-28.6] n=13 | 11.4[3.2-40.2] n=13 | 0 | 0.8[0.2-2.9] n=3 | 1.0[0.2-4.4] n=2 | 0 | 0.5[0.1-3.8] n=1 | 0.7[0.1-5.2] n=1 | 0 |
| **malignant tumor**  **N=3** | 0.9[0.1-10.3] n=1 | 1.2[0.1-13.2] n=1 | 20.0[1.8-220.9] n=2 | 0 | 0 | 0 | 0 | 1.8[0.2-19.6] n=1 | 0 | 0 | 15.1[1.4-166.8] n=2 | 20.6[1.9-227.6] n=2 | 0 |
| **neonatal cardiovascular disorder**  **N=18** | 2.3[0.9-5.9] n=10 | 1.9[0.8-4.9] n=8 | 2.0[0.6-6.9] n=3 | 1.6[0.2-12.0] n=1 | 2.3[0.9-5.9] n=10 | 1.7[0.6-4.3] n=7 | 3.3[0.7-14.4] n=2 | 1.4[0.5-3.8] n=5 | 1.4[0.4-4.9] n=3 | 0 | 0.9[0.2-4.1] n=2 | 0.6[0.1-4.5] n=1 | 1.7[0.2-12.8] n=1 |
| **neonatal coagulation disorder**  **N=10** | 0.8[0.2-3.1] n=3 | 1.0[0.3-4.0] n=3 | 0 | 0 | 1.9[0.5-6.4] n=5 | 0.3[0.0-2.3] n=1 | 2.9[0.4-23.0] n=1 | 0.9[0.2-4.2] n=2 | 0 | 0 | 0 | 0 | 0 |
| **neonatal digestive disorder**  **N=20** | 1.0[0.4-2.5] n=7 | 1.0[0.4-2.7] n=6 | 1.1[0.3-4.8] n=2 | 0 | 0.6[0.2-1.7] n=5 | 0.6[0.2-1.9] n=4 | 0 | 1.2[0.4-3.3] n=5 | 0.8[0.2-3.4] n=2 | 3.2[0.4-24.5] n=1 | 0.8[0.2-3.6] n=2 | 0.5[0.1-4.0] n=1 | 1.5[0.2-11.4] n=1 |
| **neonatal hematological disorder**  **N=103** | 1.6[1.1-2.4] n=48 | 1.6[1.1-2.4] n=41 | 2.0[1.2-3.4] n=17 | 1.7[0.7-3.9] n=6 | 3.2[2.1-4.8] n=64 | 1.7[1.1-2.5] n=40 | 0.8[0.2-2.5] n=3 | 2.1[1.4-3.2] n=38 | 0.4[0.2-1.0] n=6 | 0 | 0.6[0.3-1.3] n=8 | 0.4[0.1-1.1] n=4 | 1.8[0.8-4.2] n=6 |
| **neonatal infection**  **N=60** | 2.0[1.2-3.4] n=31 | 2.3[1.4-3.8] n=29 | 0.7[0.3-2.0] n=4 | 1.4[0.4-4.6] n=3 | 1.9[1.1-3.1] n=30 | 1.3[0.8-2.2] n=20 | 1.4[0.4-4.4] n=3 | 0.9[0.5-1.7] n=12 | 0.2[0.1-1.0] n=2 | 1.0[0.1-7.5] n=1 | 1.0[0.4-2.2] n=7 | 0.9[0.4-2.3] n=5 | 1.0[0.2-4.1] n=2 |
| **neonatal metabolic disorder**  **N=30** | 1.2[0.6-2.6] n=12 | 0.9[0.4-2.0] n=8 | 2.0[0.8-5.3] n=5 | 3.0[0.9-10.1] n=3 | 1.2[0.6-2.6] n=12 | 0.6[0.3-1.6] n=6 | 2.9[0.9-9.8] n=3 | 1.1[0.5-2.5] n=7 | 1.1[0.4-3.1] n=4 | 0 | 1.2[0.4-3.3] n=4 | 0.7[0.2-3.1] n=2 | 2.1[0.5-8.7] n=2 |
| **neonatal neuronal disorder**  **N=46** | 1.0[0.5-1.8] n=16 | 1.2[0.6-2.2] n=15 | 4.0[2.1-7.8] n=13 | 4.2[1.7-10.1] n=6 | 1.0[0.5-1.8] n=16 | 0.5[0.3-1.2] n=8 | 1.8[0.6-6.0] n=3 | 1.7[0.9-3.2] n=15 | 1.5[0.7-3.2] n=8 | 1.3[0.2-9.9] n=1 | 2.7[1.4-5.3] n=12 | 1.9[0.8-4.2] n=7 | 3.6[1.4-9.3] n=5 |
| **neonatal respiratory disorder**  **N=170** | 1.2[0.9-1.7] n=67 | 1.3[1.0-1.8] n=60 | 2.4[1.6-3.5] n=31 | 2.2[1.2-4.0] n=12 | 1.9[1.4-2.6] n=85 | 1.6[1.1-2.2] n=63 | 1.3[0.6-2.7] n=8 | 2.4[1.8-3.4] n=67 | 1.8[1.2-2.7] n=34 | 0.7[0.2-2.9] n=2 | 1.1[0.7-1.8] n=22 | 1.2[0.7-2.0] n=18 | 0.7[0.2-1.9] n=4 |
| **neonatal sensory disorder**  **N=5** | 1.2[0.2-7.4] n=2 | 0.6[0.1-5.4] n=1 | 0 | 0 | 7.5[0.8-66.8] n=4 | 1.7[0.3-10.4] n=2 | 0 | 5.3[0.9-31.9] n=3 | 0 | 0 | 0 | 0 | 0 |
| **pregnancy complication**  **N=598** | 1.6[1.3-1.9] n=261 | 1.3[1.1-1.6] n=205 | 1.6[1.2-2.1] n=77 | 2.8[1.9-4.1] n=44 | 2.0[1.6-2.4] n=288 | 1.7[1.4-2.1] n=223 | 1.5[1.0-2.3] n=30 | 1.1[0.9-1.3] n=139 | 0.7[0.5-0.9] n=56 | 1.8[1.0-3.2] n=15 | 3.9[3.1-4.9] n=159 | 3.7[2.9-4.8] n=121 | 3.1[2.1-4.6] n=44 |
| **chorioamnionitis**  **N=8** | 0.3[0.0-2.2] n=1 | 0 | 0 | 0 | 0.3[0.0-2.2] n=1 | 0.4[0.0-3.0] n=1 | 0 | 2.1[0.5-8.9] n=3 | 1.0[0.1-8.2] n=1 | 0 | 0 | 0 | 0 |
| **Eclampsia**  **N=3** | 0.9[0.1-10.3] n=1 | 1.2[0.1-13.2] n=1 | 0 | 0 | 0.9[0.1-10.3] n=1 | 0 | 13.1[1.2-144.9] n=1 | 1.8[0.2-19.6] n=1 | 3.5[0.3-38.9] n=1 | 0 | 0 | 0 | 0 |
| **gestational diabetes**  **N=13** | 0.6[0.2-2.0] n=3 | 0.7[0.2-2.6] n=3 | 1.8[0.4-8.2] n=2 | 2.2[0.3-17.4] n=1 | 0.6[0.2-2.0] n=3 | 0.8[0.2-2.8] n=3 | 0 | 1.6[0.5-5.1] n=4 | 0 | 11.3[2.5-52.5] n=2 | 3.4[1.0-11.0] n=4 | 4.6[1.4-15.0] n=4 | 0 |
| **HELLP syndrome**  **N=3** | 3.7[0.3-41.1] n=2 | 4.8[0.4-52.9] n=2 | 0 | 0 | 0.9[0.1-10.3] n=1 | 0 | 13.1[1.2-144.9] n=1 | 0 | 0 | 0 | 3.8[0.3-41.6] n=1 | 0 | 14.4[1.3-160.0] n=1 |
| **hydrops fetalis**  **N=6** | 0 | 0 | 2.0[0.2-17.1] n=1 | 5.4[0.6-46.6] n=1 | 0 | 0 | 0 | 0 | 0 | 0 | 0 | 0 | 0 |
| **hypertension/pre-eclampsia**  **N=84** | 1.6[1.0-2.4] n=38 | 1.7[1.1-2.7] n=35 | 0.9[0.4-2.0] n=7 | 1.7[0.7-4.3] n=5 | 1.6[1.0-2.4] n=38 | 1.9[1.2-2.9] n=35 | 0 | 0.4[0.2-0.8] n=9 | 0.3[0.1-0.9] n=4 | 2.3[0.7-7.5] n=3 | 4.9[3.1-7.8] n=32 | 5.2[3.3-8.4] n=27 | 1.8[0.7-4.6] n=5 |
| **intrauterine growth restriction**  **N=327** | 2.0[1.6-2.5] n=162 | 1.5[1.2-1.9] n=123 | 2.8[2.1-3.8] n=64 | 4.3[2.9-6.5] n=36 | 2.5[2.0-3.1] n=179 | 2.1[1.7-2.7] n=141 | 1.2[0.7-2.1] n=14 | 1.6[1.3-2.1] n=99 | 0.9[0.6-1.3] n=37 | 2.1[1.1-4.2] n=10 | 2.9[2.2-3.8] n=82 | 2.8[2.0-3.8] n=62 | 2.8[1.7-4.4] n=25 |
| **Oligohydramnios**  **N=139** | 1.5[1.1-2.1] n=62 | 1.0[0.7-1.5] n=42 | 0.5[0.2-1.1] n=7 | 1.0[0.4-2.5] n=5 | 1.5[1.1-2.1] n=62 | 1.3[0.9-1.9] n=47 | 2.6[1.4-4.8] n=12 | 0.6[0.4-0.9] n=20 | 0.5[0.2-0.9] n=9 | 0 | 4.7[3.3-6.8] n=50 | 3.5[2.3-5.2] n=33 | 4.9[2.8-8.3] n=18 |
| **Polyhydramnios**  **N=9** | 0.2[0.0-1.9] n=1 | 0.3[0.0-2.4] n=1 | 0 | 0 | 0.2[0.0-1.9] n=1 | 0 | 3.3[0.4-26.2] n=1 | 0.4[0.1-3.5] n=1 | 0.9[0.1-7.0] n=1 | 0 | 0.9[0.1-7.5] n=1 | 0 | 3.6[0.4-29.0] n=1 |
| **pregnancy hemorrhage**  **N=9** | 0.5[0.1-2.6] n=2 | 0.7[0.1-3.3] n=2 | 1.2[0.2-10.0] n=1 | 3.4[0.4-27.2] n=1 | 0.5[0.1-2.6] n=2 | 0 | 0 | 2.8[0.8-10.6] n=4 | 0.9[0.1-7.0] n=1 | 0 | 0 | 0 | 0 |
| **threatened preterm labor**  **N=5** | 1.2[0.2-7.4] n=2 | 1.6[0.3-9.6] n=2 | 0 | 0 | 1.2[0.2-7.4] n=2 | 1.7[0.3-10.4] n=2 | 0 | 0 | 0 | 0 | 1.9[0.2-16.9] n=1 | 2.6[0.3-23.0] n=1 | 0 |
| **preterm birth**  **N=807** | 2.4[2.0-2.8] n=409 | 2.4[2.0-2.8] n=355 | 2.0[1.6-2.6] n=115 | 1.8[1.2-2.6] n=43 | 3.6[3.0-4.2] n=465 | 3.3[2.8-3.9] n=382 | 1.4[1.0-2.1] n=38 | 1.9[1.6-2.3] n=250 | 2.1[1.7-2.6] n=157 | 0.5[0.3-1.1] n=8 | 1.5[1.2-1.9] n=124 | 1.6[1.3-2.1] n=99 | 1.3[0.8-1.9] n=32 |
| **fetal death – stillbirth**  **N=139** | 1.4[1.0-2.0] n=59 | 0.7[0.5-1.1] n=33 | 0.7[0.3-1.3] n=9 | 0.2[0.0-1.4] n=1 | 3.1[2.2-4.4] n=85 | 2.4[1.7-3.4] n=65 | 0.6[0.2-1.8] n=3 | 1.1[0.7-1.7] n=33 | 0.3[0.1-0.6] n=5 | 0 | 0.3[0.1-0.7] n=5 | 0.3[0.1-0.8] n=4 | 0.2[0.0-1.4] n=1 |

**Table S11(bis):**

| **ADR** | **vinca alcaloid**  **N=468** | **Vinorelbine**  **N=27** | **HER2 inhibitor**  **N=317** | **Trastuzumab**  **N=291** | **Pertuzumab**  **N=57** | **trastuzumab emtansine**  **N=21** | **Lapatinib**  **N=13** | **Tucatinib**  **N=2** | **Immunotherapy**  **N=153** | **Pembrolizumab**  **N=36** | **CDK inhibitor**  **N=13** | **Palbociclib**  **N=9** | **Ribociclib**  **N=3** | **PI3K-AKT-mTOR inhibitor**  **N=6** | **Everolimus**  **N=4** |
| --- | --- | --- | --- | --- | --- | --- | --- | --- | --- | --- | --- | --- | --- | --- | --- |
| **congenital malformation**  **N=223** | 1.3[0.9-1.9] n=39 | 3.2[1.2-8.5] n=5 | 1.7[1.2-2.5] n=33 | 1.7[1.1-2.5] n=30 | 1.6[0.7-3.9] n=6 | 1.5[0.3-6.3] n=2 | 1.2[0.1-8.9] n=1 | 13.9[0.9-223.0] n=1 | 1.0[0.5-1.9] n=10 | 0 | 1.2[0.1-8.9] n=1 | 0 | 6.9[0.6-76.9] n=1 | 0 | 0 |
| **cardiovascular malformation**  **N=81** | 2.0[1.2-3.4] n=20 | 5.1[1.5-17.4] n=3 | 1.3[0.7-2.6] n=10 | 1.3[0.6-2.6] n=9 | 2.3[0.7-7.4] n=3 | 4.3[1.0-18.7] n=2 | 0 | 0 | 0.8[0.2-2.5] n=3 | 0 | 3.4[0.4-26.1] n=1 | 0 | 20.2[1.8-224.7] n=1 | 0 | 0 |
| **digestive malformation**  **N=19** | 2.2[0.8-6.1] n=5 | 15.4[3.4-70.1] n=2 | 0 | 0 | 0 | 0 | 0 | 0 | 1.1[0.2-8.7] n=1 | 0 | 0 | 0 | 0 | 0 | 0 |
| **face malformation**  **N=18** | 1.2[0.4-4.2] n=3 | 16.3[3.6-74.8] n=2 | 1.2[0.3-5.2] n=2 | 1.3[0.3-5.7] n=2 | 0 | 0 | 0 | 0 | 0 | 0 | 0 | 0 | 0 | 0 | 0 |
| **fetal malformation NOS**  **N=29** | 1.6[0.6-3.9] n=6 | 9.6[2.2-42.8] n=2 | 3.0[1.3-7.2] n=7 | 2.7[1.1-6.8] n=6 | 0 | 0 | 0 | 117.1[7.1-1919.9] n=1 | 2.4[0.7-8.0] n=3 | 0 | 0 | 0 | 0 | 0 | 0 |
| **genetic disorder**  **N=13** | 0 | 0 | 4.2[1.3-13.8] n=4 | 4.7[1.4-15.2] n=4 | 4.8[0.6-37.7] n=1 | 0 | 0 | 0 | 0 | 0 | 0 | 0 | 0 | 0 | 0 |
| **genitourinary malformation**  **N=21** | 1.4[0.5-4.3] n=4 | 0 | 1.6[0.5-5.4] n=3 | 1.7[0.5-5.9] n=3 | 6.2[1.4-27.2] n=2 | 0 | 0 | 0 | 2.2[0.5-9.5] n=2 | 0 | 0 | 0 | 0 | 0 | 0 |
| **multiple malformation**  **N=8** | 0.9[0.1-7.1] n=1 | 0 | 0 | 0 | 0 | 0 | 0 | 0 | 0 | 0 | 0 | 0 | 0 | 0 | 0 |
| **musculoskeletal malformation**  **N=40** | 1.1[0.4-2.6] n=6 | 0 | 0.5[0.1-2.1] n=2 | 0.3[0.0-1.9] n=1 | 0 | 0 | 7.0[0.9-54.9] n=1 | 0 | 3.0[1.2-7.8] n=5 | 0 | 0 | 0 | 0 | 0 | 0 |
| **neurological malformation**  **N=23** | 0.9[0.3-3.1] n=3 | 0 | 0.4[0.1-3.2] n=1 | 0.5[0.1-3.5] n=1 | 0 | 0 | 0 | 0 | 0 | 0 | 0 | 0 | 0 | 0 | 0 |
| **respiratory tract malformation**  **N=9** | 0 | 0 | 19.2[4.8-77.3] n=6 | 21.2[5.3-85.1] n=6 | 16.9[3.4-83.0] n=2 | 0 | 0 | 0 | 0 | 0 | 0 | 0 | 0 | 0 | 0 |
| **sensory defect**  **N=24** | 0 | 0 | 1.4[0.4-4.6] n=3 | 1.5[0.4-5.0] n=3 | 0 | 0 | 0 | 0 | 0 | 0 | 0 | 0 | 0 | 0 | 0 |
| **neonatal complication**  **N=402** | 2.2[1.7-2.8] n=98 | 1.3[0.4-3.7] n=4 | 1.0[0.7-1.4] n=38 | 1.1[0.7-1.5] n=37 | 0.4[0.1-1.3] n=3 | 0.8[0.2-3.3] n=2 | 0 | 0 | 0.6[0.3-1.1] n=12 | 0.4[0.1-1.8] n=2 | 0.6[0.1-4.6] n=1 | 0.9[0.1-7.2] n=1 | 0 | 0 | 0 |
| **benign tumor**  **N=9** | 0 | 0 | 0 | 0 | 0 | 0 | 0 | 0 | 0 | 0 | 0 | 0 | 0 | 0 | 0 |
| **Hyperbilirubinemia**  **N=39** | 1.3[0.6-3.0] n=7 | 0 | 0.5[0.1-2.1] n=2 | 0.3[0.0-2.0] n=1 | 0 | 4.3[0.6-32.7] n=1 | 0 | 0 | 0.5[0.1-4.0] n=1 | 0 | 0 | 0 | 0 | 0 | 0 |
| **immune deficiency**  **N=16** | 10.3[3.7-28.5] n=10 | 0 | 0 | 0 | 0 | 0 | 0 | 0 | 0 | 0 | 0 | 0 | 0 | 0 | 0 |
| **malignant tumor**  **N=3** | 3.0[0.3-33.6] n=1 | 0 | 0 | 0 | 0 | 0 | 0 | 0 | 0 | 0 | 0 | 0 | 0 | 0 | 0 |
| **neonatal cardiovascular disorder**  **N=18** | 3.9[1.5-10.1] n=7 | 0 | 1.2[0.3-5.2] n=2 | 1.3[0.3-5.7] n=2 | 0 | 0 | 0 | 0 | 0 | 0 | 0 | 0 | 0 | 0 | 0 |
| **neonatal coagulation disorder**  **N=10** | 1.5[0.3-7.2] n=2 | 0 | 0 | 0 | 0 | 0 | 0 | 0 | 0 | 0 | 0 | 0 | 0 | 0 | 0 |
| **neonatal digestive disorder**  **N=20** | 2.6[1.0-6.9] n=6 | 6.6[0.9-51.2] n=1 | 1.7[0.5-5.7] n=3 | 1.8[0.5-6.3] n=3 | 0 | 0 | 0 | 0 | 1.1[0.1-8.2] n=1 | 0 | 0 | 0 | 0 | 0 | 0 |
| **neonatal hematological disorder**  **N=103** | 4.3[2.9-6.5] n=41 | 2.5[0.6-10.8] n=2 | 0.3[0.1-0.9] n=3 | 0.3[0.1-1.0] n=3 | 0 | 0 | 0 | 0 | 0.4[0.1-1.6] n=2 | 0 | 0 | 0 | 0 | 0 | 0 |
| **neonatal infection**  **N=60** | 3.9[2.3-6.7] n=23 | 4.4[1.0-19.2] n=2 | 1.3[0.6-2.8] n=7 | 1.4[0.6-3.1] n=7 | 1.0[0.1-7.1] n=1 | 2.7[0.4-20.7] n=1 | 0 | 0 | 0.3[0.0-2.5] n=1 | 0 | 0 | 0 | 0 | 0 | 0 |
| **neonatal metabolic disorder**  **N=30** | 1.5[0.6-3.8] n=6 | 0 | 0.3[0.0-2.4] n=1 | 0.4[0.0-2.6] n=1 | 0 | 0 | 0 | 0 | 2.3[0.7-7.7] n=3 | 3.2[0.4-24.1] n=1 | 0 | 0 | 0 | 0 | 0 |
| **neonatal neuronal disorder**  **N=46** | 0.7[0.3-1.9] n=5 | 9.4[2.7-32.5] n=3 | 1.2[0.5-2.9] n=5 | 1.3[0.5-3.2] n=5 | 0 | 0 | 0 | 0 | 0.5[0.1-3.3] n=1 | 0 | 0 | 0 | 0 | 0 | 0 |
| **neonatal respiratory disorder**  **N=170** | 2.0[1.4-2.9] n=41 | 2.3[0.7-7.8] n=3 | 1.4[0.9-2.3] n=22 | 1.6[1.0-2.5] n=22 | 0 | 0 | 0 | 0 | 0.9[0.4-1.9] n=7 | 0.5[0.1-3.9] n=1 | 0 | 0 | 0 | 0 | 0 |
| **neonatal sensory disorder**  **N=5** | 9.2[1.5-55.0] n=3 | 0 | 0 | 0 | 0 | 0 | 0 | 0 | 5.2[0.6-46.7] n=1 | 0 | 0 | 0 | 0 | 0 | 0 |
| **pregnancy complication**  **N=598** | 1.6[1.3-2.0] n=115 | 5.8[2.7-12.4] n=15 | 2.3[1.8-2.9] n=99 | 2.4[1.8-3.1] n=94 | 1.3[0.7-2.5] n=13 | 0.8[0.2-2.6] n=3 | 3.9[1.3-11.7] n=6 | 0 | 0.8[0.5-1.2] n=23 | 0.1[0.0-0.9] n=1 | 0.4[0.0-2.9] n=1 | 0.6[0.1-4.5] n=1 | 0 | 2.3[0.4-12.4] n=2 | 1.5[0.2-14.6] n=1 |
| **chorioamnionitis**  **N=8** | 0.9[0.1-7.1] n=1 | 0 | 0 | 0 | 0 | 0 | 0 | 0 | 0 | 0 | 0 | 0 | 0 | 0 | 0 |
| **Eclampsia**  **N=3** | 0 | 0 | 0 | 0 | 0 | 0 | 0 | 0 | 0 | 0 | 0 | 0 | 0 | 0 | 0 |
| **gestational diabetes**  **N=13** | 1.1[0.2-5.0] n=2 | 0 | 2.9[0.8-10.4] n=3 | 3.1[0.9-11.5] n=3 | 0 | 0 | 0 | 0 | 3.8[0.8-17.2] n=2 | 0 | 0 | 0 | 0 | 0 | 0 |
| **HELLP syndrome**  **N=3** | 0 | 0 | 0 | 0 | 0 | 0 | 0 | 0 | 10.4[0.9-115.1] n=1 | 0 | 0 | 0 | 0 | 0 | 0 |
| **hydrops fetalis**  **N=6** | 0 | 0 | 0 | 0 | 0 | 0 | 0 | 0 | 0 | 0 | 0 | 0 | 0 | 0 | 0 |
| **hypertension/pre-eclampsia**  **N=84** | 1.2[0.7-2.2] n=14 | 0 | 0.3[0.1-1.1] n=3 | 0.2[0.1-1.0] n=2 | 0.7[0.1-5.0] n=1 | 4.1[0.9-18.0] n=2 | 0 | 0 | 1.9[0.9-4.2] n=7 | 0 | 0 | 0 | 0 | 0 | 0 |
| **intrauterine growth restriction**  **N=327** | 1.8[1.3-2.4] n=70 | 1.6[0.5-4.6] n=4 | 0.9[0.6-1.4] n=29 | 0.9[0.6-1.3] n=26 | 0.9[0.3-2.2] n=5 | 0 | 7.9[2.7-23.8] n=6 | 0 | 0.8[0.4-1.4] n=12 | 0.3[0.0-1.9] n=1 | 0 | 0 | 0 | 4.6[0.8-25.1] n=2 | 3.0[0.3-29.4] n=1 |
| **Oligohydramnios**  **N=139** | 1.4[0.9-2.2] n=26 | 14.4[6.5-32.0] n=10 | 12.8[9.0-18.3] n=72 | 14.0[9.8-20.1] n=71 | 3.9[1.8-8.4] n=8 | 1.1[0.2-8.6] n=1 | 0 | 0 | 0.1[0.0-1.0] n=1 | 0 | 0 | 0 | 0 | 0 | 0 |
| **Polyhydramnios**  **N=9** | 0 | 0 | 2.7[0.6-13.1] n=2 | 3.0[0.6-14.4] n=2 | 0 | 0 | 0 | 0 | 0 | 0 | 0 | 0 | 0 | 0 | 0 |
| **pregnancy hemorrhage**  **N=9** | 3.0[0.8-12.2] n=3 | 15.7[1.9-130.4] n=1 | 1.2[0.1-9.5] n=1 | 1.3[0.2-10.4] n=1 | 0 | 0 | 0 | 0 | 0 | 0 | 0 | 0 | 0 | 0 | 0 |
| **threatened preterm labor**  **N=5** | 1.5[0.2-13.6] n=1 | 0 | 0 | 0 | 0 | 0 | 0 | 0 | 0 | 0 | 0 | 0 | 0 | 0 | 0 |
| **preterm birth**  **N=807** | 2.7[2.2-3.4] n=200 | 2.5[1.2-5.4] n=12 | 0.6[0.4-0.8] n=50 | 0.6[0.4-0.8] n=46 | 0.3[0.1-0.7] n=5 | 0.5[0.2-1.8] n=3 | 0.9[0.3-3.4] n=3 | 0 | 0.8[0.5-1.2] n=31 | 0.2[0.0-0.8] n=2 | 0.6[0.1-2.5] n=2 | 0.4[0.0-3.1] n=1 | 1.6[0.1-17.1] n=1 | 0 | 0 |
| **fetal death – stillbirth**  **N=139** | 3.5[2.4-5.0] n=48 | 0 | 0.3[0.1-0.7] n=4 | 0.2[0.1-0.7] n=3 | 0 | 0 | 1.9[0.2-14.8] n=1 | 0 | 0.8[0.3-1.9] n=5 | 0 | 0 | 0 | 0 | 0 | 0 |

## Table S12. Missing data per variable in Table 1 (N total = 3,310 reports).

*Numbers in Table 1 do not always sum to 3,310 due to missing data for individual variables. The table below reports the number of reports with missing information for each variable, stratified by exposure group. Missing data were not imputed; reports with missing values for a given variable were excluded from the denominator for that variable only.*

| **Variable** | **Overall missing n (%)** | **BC treatment group missing n (%)** | **Other anticancer group missing n (%)** | **Reason for missingness** |
| --- | --- | --- | --- | --- |
| Region | 1 (0.03%) | 0 (0%) | 1 (0.07%) | Country not recorded in report |
| Notifier type | 212 (6.4%) | 132 (7.4%) | 80 (5.3%) | Notifier identity not recorded in report |
| Number of notifiers | 212 (6.4%) | 132 (7.4%) | 80 (5.3%) | Same reports as notifier type (notifier field not recorded) |
| Report context (clinical trial / routine care) | 1 (0.03%) | 0 (0%) | 1 (0.07%) | Report type not specified |
| Cancer type | 474 (14.3%) | 226 (12.6%) | 248 (16.3%) | No cancer diagnosis identifiable in the report (cancer type not mentioned) |
| Age | 0 (0%) | 0 (0%) | 0 (0%) | Complete for all reports |
| Year of first report | 0 (0%) | 0 (0%) | 0 (0%) | Complete for all reports (most recent: 2023) |
| Number of suspect/interacting drugs | 0 (0%) | 0 (0%) | 0 (0%) | Complete for all reports (derived from drug listing in report) |

*BC, breast cancer. Percentages for missingness are calculated on the total number of reports in each group (overall n=3,310; BC treatment group n=1,789; other anticancer group n=1,521). Note: cancer type proportions in Table 1 are calculated on reports with available cancer data only (n=2,836), which explains why the percentages in Table 1 sum to 100% within that subgroup rather than across all 3,310 reports.*

# Supplemental Methods

## Identification of reports addressing mother vs fetal/newborn

### Position of the problem

To better assess adverse outcomes. we attributed each report to the mother or the fetus/child. Attribution was made using a scoring system.

### Scoring system for each report

| **Variable** | **Modality** | **Mother score** | **Fetal/newborn score** |
| --- | --- | --- | --- |
| Age group | Neonate. child, infant |  | 2 |
|  | Adult | 2 |  |
| Age unit | Days, day, months, month, hour, hours, weeks |  | 2 |
| Age | <10 |  | 1 |
| Age unit  Age | Age unit = year  & Age >10 | 2 |  |
| Weight | <10 |  | 1 |
|  | >10 | 1 |  |
| Sex | Male |  | 1 |
| Reports seriousness criteria | Congenital anomaly/birth defect |  |  |
| Drug route administration | Transplacental |  | 2 |
| Reaction PT | "Drug exposure in utero",  "Foetal exposure during pregnancy",  "Foetal exposure during delivery",  "Foetal exposure timing unspecified" |  | 1 |
| Reaction PT | "Drug exposure before pregnancy",  "Maternal exposure before pregnancy",  "Exposure during pregnancy",  "First trimester pregnancy",  "High risk pregnancy",  "Maternal exposure during delivery",  "Maternal exposure during pregnancy",  "Pregnancy",  "Pregnant",  "Pregnancy on contraceptive",  "Pregnancy on oral contraceptive",  "Pregnancy with advanced maternal age",  "Pregnancy with contraceptive device",  "Pregnancy with injectable contraceptive",  "Unintended pregnancy",  "Unwanted pregnancy",  "Maternal exposure timing unspecified" | 1 |  |
| Reaction PT | PT, HLT or HLGT code for a malignant tumor (SOC = "Neoplasms benign, malignant and unspecified (incl cysts and polyps)", terms include lymphoma, leukemia, myeloma, malignant, malignancy, sarcoma, carcinoma, “metast” | 1 |  |


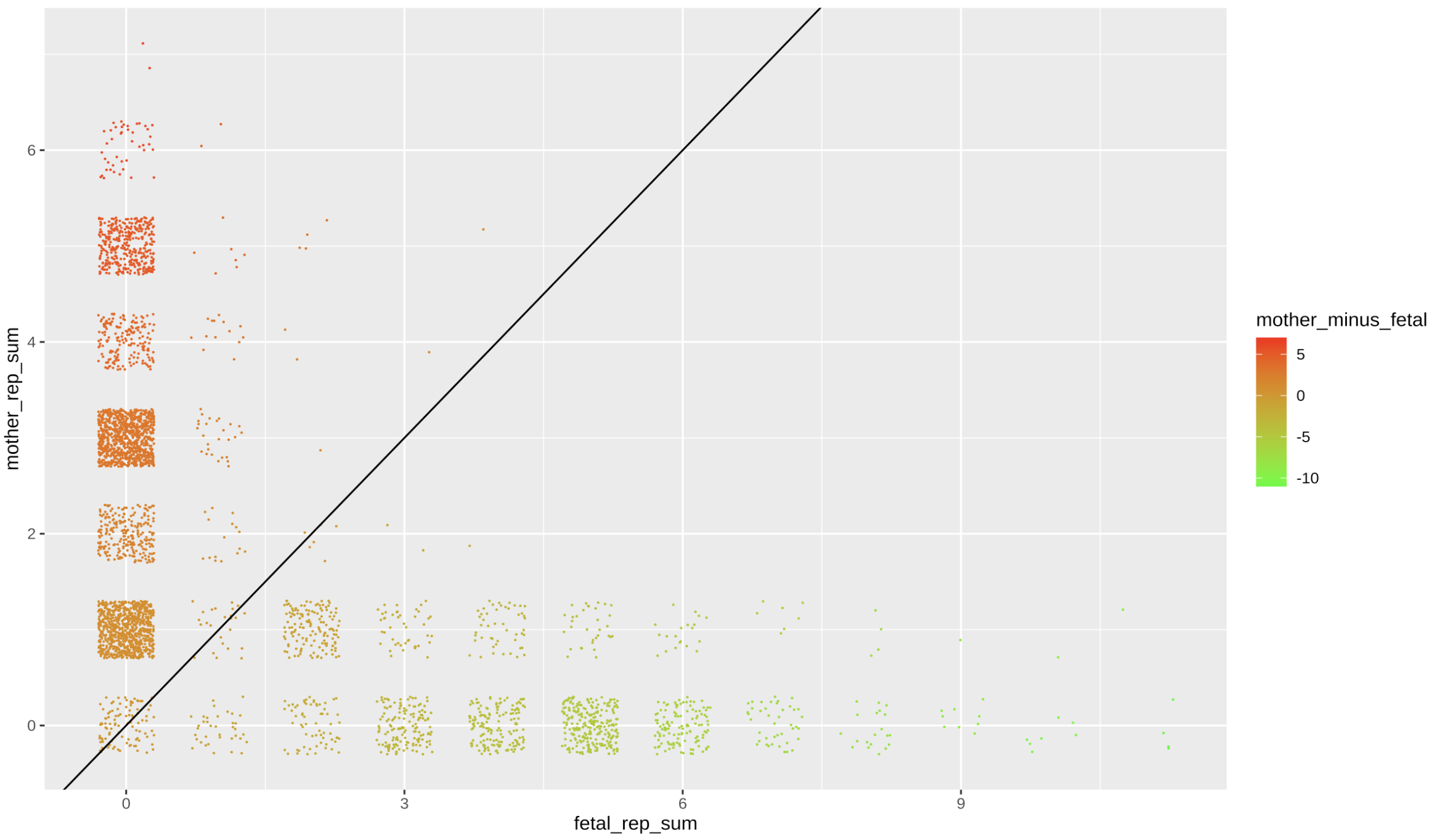


Figure: Difference of the score for maternal items (mother_rep_score) with fetal/newborn items (fetal_rep_sum)

## Final attribution

Reports with “score mother” > “score fetal/newborn” were attributed to the mother and reports with “score mother” < “score fetal/newborn” were attributed to the fetus/newborn.

132 reports on 3977 (3.3%) screened had a “score mother” = “score fetal/newborn” and were deciphered individually.

## Deduplication algorithm

### Problem position

Due to the possibility of reporting from different sources inherent to pharmacovigilance, there is an important possibility than some cases could be reported multiple times in VigiBase. To address this problem, the Upsala Monitoring Center, within the “VigiLyze” platform, integrated a duplication detection algorithm, “VigiMatch”. However, from our experience, and particularly in the VITALITY cohort, there remains a risk that some cases could still be reported multiple times, notably because some reports are also extracted from literature.

To address this problem, we developed an in-house information entropy-based algorithm of detection of duplicates.

### Initial selection

First, we selected all reports for which we found:

1. Same country origin
2. Same anticancer drugs declared within report
3. Same cancer identified in report
4. Report addressing mother or fetus/newborn

Then, all the tandems of potential duplicates were evaluated.

Information entropy

For each variable considered, i.e. country of report, we evaluated the amount of information brought by the report using an entropy-based scoring system.

For each tandem, we calculated an entropy-based score of the similarities of reports.

Entropy score for each variable was calculated with the following formula:

$$\frac{Ntot}{n}. log(\frac{Ntot}{n})$$

Were:

- **Ntot** is the total number of total reports in the cohort
- **n** is the number of occurrences of the variable

For example, a tandem with France as the same country had a “country” entropy score of:

n=151 reports from France and N_tot_ is 3956

S_country_ = 3956/151.log(3956/151) = 85.6

Total entropy score was calculated from the entropy of the following variable:

- country
- age
- weight
- size
- mother age
- mother size
- mother weight
- date of last menstruation
- cancer type
- year of first report
- year of first event reported
- drugs
- start date of drugs
- reported and MedDRA PT-mapped adverse event
- start date of adverse events
- mapped materno-fetal adverse events

for drugs and adverse events, each match was counted individually

Score validation and determination of entropy score threshold using reports which were also reported in the literature and for which the reference was available within the CIOMS extracted file, and for whom the status of duplicate was unambiguous, we found that a threshold of 2000 was had a specificity of 100%.


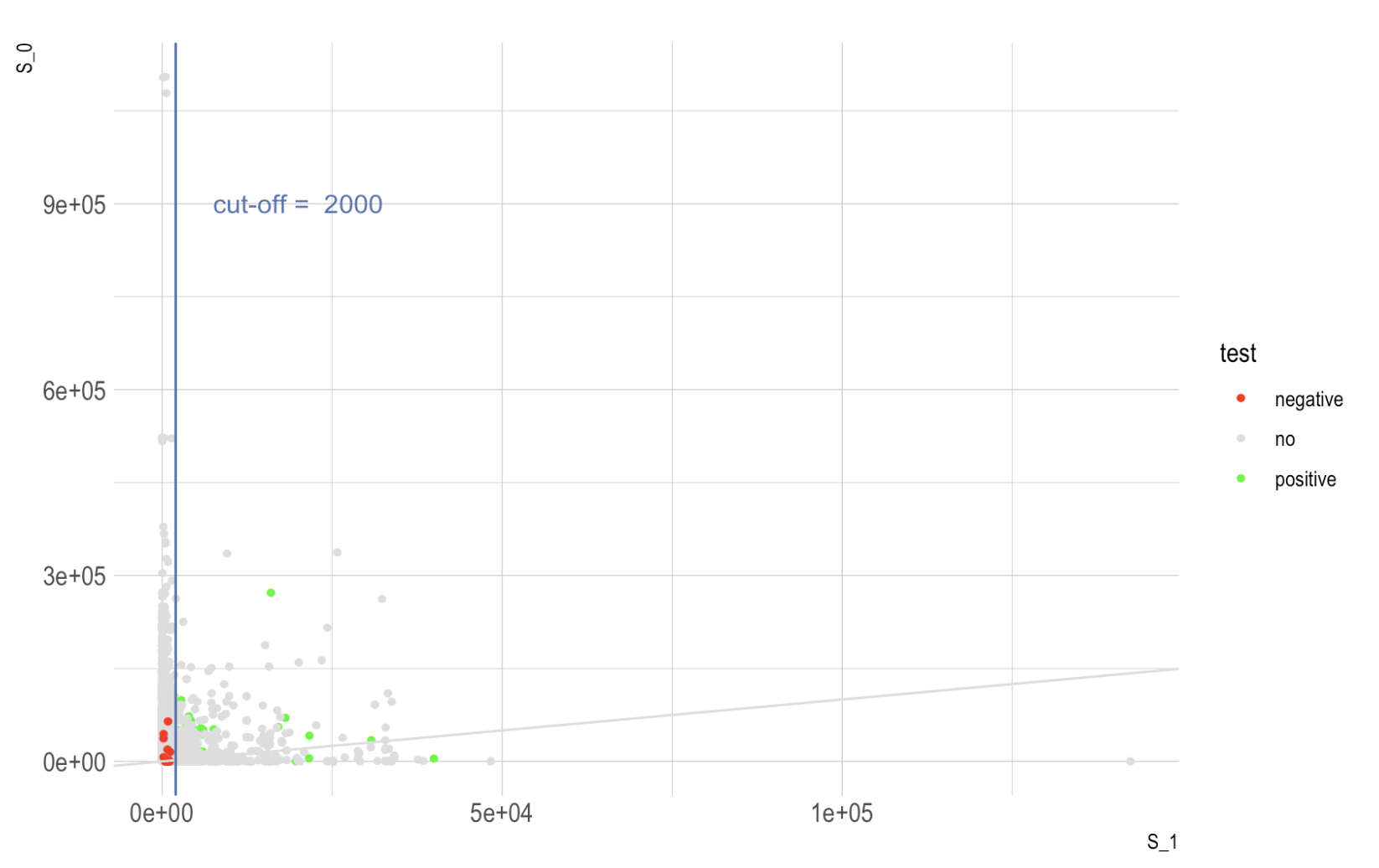


Figure. Entropy score S_1 and score for discordance S_0 for 46,159 tandems of reports from 3,956 reports within the cohort. Test were performed on reports that had a literature reference within the CIOMS extracted case.

Of note, same reports could have a discordance entropy elevated when additional and rarely reports information are present in one of the duplicates and not the other one.

The algorithm was looped until no more duplicated was detected.

### Fusion of duplicates

After detection of duplicates, information from reports were mixed within a single report using available data for all demographic data and drugs and adverse events. For demographic data, whenever there was a discrepancy, data from the latest reports was kept.

## Fusion of dyads

### Position of the problem

In some situations, for a single case, 2 reports could be declared for both the fetus/newborn and the mother. This present two problems: first, the denominator is modified. Second, for materno-fetal toxicity such as hydrops fetalis, both the mother and the child could be declared individually, amplifying a fake signal.

Initial selection of dyads

First, we selected all reports for which we found:

### Inclusion criteria

1. Same country origin
2. Same anticancer drugs declared within report
3. Same cancer identified in report
4. Date of first declaration with a latitude of 1 year
5. Date of first event with a latitude of 1 year
6. One of the reports is addressing the mother and the second is addressing the fetus/newborn

### Exclusion criteria

- different last menstruation date

- different mother weight size and age

- dyads from published literature not compatible with dyads

Then, all the tandems of potential dyads were evaluated using entropy score.

An entropy score of 500 was deemed relevant. Dyads were merged and interpreted as one single report.
